# Genome-wide identification of grape ANS gene family and expression analysis at different fruit coloration stages

**DOI:** 10.1186/s12870-023-04648-3

**Published:** 2023-12-09

**Authors:** Yongqing Feng, Xuechun Tian, Wei Liang, XinTong Nan, Aoning Zhang, Wenfang Li, Zonghuan Ma

**Affiliations:** College of Horticulture, Gansu Agricultural University, Lanzhou, 730070 People’s Republic of China

**Keywords:** Grape, VvANS gene family, Different coloring stages, Expression analysis

## Abstract

**Background:**

Anthocyanin synthase (ANS) is the enzyme downstream of the anthocyanins synthesis pathway and the rate-limiting enzyme of the synthesis pathway. It catalyzes the conversion of colorless anthocyanins to anthocyanins and plays an important role in plant color presentation and stress resistance. However, *ANS* gene is rarely studied in grapes.

**Results:**

In this study, 121 *VvANS* genes were identified and distributed on 18 chromosomes, VvANS family members were divided into 8 subgroups. Secondary structure prediction showed mainly irregular coils and α-helices, and subcellular localization indicated that VvANS gene family is mainly located in chloroplast, cytoplasm and nucleus. The promoter region of the VvANS gene family contains multiple cis-acting elements that are associated with light, abiotic stress, and hormones. Intraspecific collinearity analysis showed that there were 13 pairs of collinearity between *VvANS* genes. Interspecific collinearity analysis showed that there was more collinearity between grape, apple and *Arabidopsis*, but less collinearity between grape and rice. Microarray data analysis showed that *VvANS17*, *VvANS23* and *VvANS75* had higher expression levels in flesh and peel, while *VvANS25*, *VvANS64* and *VvANS106* had higher expression levels in flower. The results of qRT-PCR analysis showed that *VvANS* genes were expressed throughout the whole process of fruit coloring, such as *VvANS47* and *VvANS55* in the green fruit stage, *VvANS3*, *VvANS64* and *VvANS90* in the initial fruit color turning stage. The expression levels of *VvANS21*, *VvANS79* and *VvANS108* were higher at 50% coloring stage, indicating that these genes play an important role in the fruit coloring process. *VvANS4*, *VvANS66* and *VvANS113* had the highest expression levels in the full maturity stage.

**Conclusions:**

These results indicated that different members of VvANS gene family played a role in different coloring stages, and this study laid a foundation for further research on the function of ANS gene family.

**Supplementary Information:**

The online version contains supplementary material available at 10.1186/s12870-023-04648-3.

## Background

Grape is one of the most productive fruits in the world, has a long history of cultivation, with rich nutritional value, loved by consumers [1]. Grapes have a variety of benefits to the human body, such as antioxidants, anti-inflammatory, anti-cancer, anti-aging and liver protection functions [2, 3], and many processed by-products have high phenolic substances, such as wine, grape juice, raisins, etc., research found that raisins can be used to treat constipation and thirst [4].

Flavonoids are the most abundant polyphenols in plants and a class of large secondary metabolites, which are widely found in fruits and vegetables. Flavonoids can be categorized into flavonols, flavones, isoflavones, anthocyanidins, flavanones, flavanols, and chalcones based on their chemical structure [5]. With the deepening of research on flavonoids, it has been found that they have a wide range of biological activities, including antioxidant, anti-inflammatory, anti-tumor, cardiovascular protection, antiviral, liver protection and immunomodulatory activities [6–9].

Anthocyanin is a flavonoid that is formed after the glycosylation of anthocyanins, so that anthocyanins can be stable in the vacuole of plant cells. Anthocyanin is widely distributed in plant tissues, endowing roots, stems, leaves, flowers, fruits and other organs with different degrees of pink, red, orange, blue, purple and other colors, is one of the important material basis of plant color [10]. Anthocyanins are mainly divided into the six major categories, pelargonidin, cornflower, peonidin, delphinidin, malvidin and petunia pigments [11]. Anthocyanins also play a vital role in protecting plants from UV radiation, enhancing plant resistance to pathogens and promoting seed dispersal [12]. Anthocyanin reduces alcohol damage to the liver [13]. Anthocyanin also has an anti-cancer and anti-diabetic function [14, 15]. Anthocyanin biosynthesis involves many structural genes essential for flavonoid biosynthesis, including phenylalanine ammonialyase, cinnamate-4-hydroxylase, 4-coumarate: coenzyme A ligase, chalcone synthase, chalcone isomerase, flavanone-3-hydroxylase, dihydroflavonol 4-reductase, anthocyanin synthase and UDP flavonoid glucosyltransferase [16].

Anthocyanin synthase (ANS) belongs to 2-ketoglutarate-dependent dioxygenase, also known as leucoanthocyanidin dioxygenase (LDOX). Its main function is to catalyze the conversion of colorless anthocyanin to colored anthocyanin [17]. Its catalytic product is the first chromogenic compound in the anthocyanin biosynthesis pathway and plays an important role in the formation of plant organ color [18]. Studies of *LDOX* in *Arabidopsis Thaliana* showed that the decrease of *LDOX* allelic mutant anthocyanins and proanthocyanidins resulted in lighter seed coat color [19]. Some studies have shown that *ANS* is expressed in red *Perilla fulescens* leaves, but not in green Perilla [20]. *Forsythia intermedia ANS* is only expressed in sepals but not in petals or anthers, resulting in the inability to synthesize anthocyanins in petals [21]. MIYAZAKI [22] adopted RNA interference technology to inhibit the *ANS* gene of blue butterfly grass (*Torenia hybrida*), and cultivated a stable genetic white flower butterfly grass. The Basil (*Ocimum basilicum*) genome was found to contain two homologous *ANS* genes, each with a loss-of-function mutation. *ObANS1* carries a single base pair insertion, resulting in frameshift, while *ObANS2* carries a missense mutation within the active site. In the parents of purple flowers, *ANS1* is functional and *ANS2* carries nonsense mutations [23]. Kim [24] found that the loss of anthocyanin synthesis in onion was due to the allelic variation of two new *ANS* genes. In *Brassica juncea*, *BjANS* were found to be involved in the biosynthesis of procyanidins and the formation of mustard seed coat color, while the lack of *BjANS* expression hindered the biosynthesis of procyanidins in the yellow seed coat, resulting in the yellow color of the seeds due to the transparent seed coat [25].

There are many reports on ANS gene family, but few are studied in grapes, especially in terms of peel coloration. In this study, we identified members of the grape ANS gene family from the plant genome database and used bioinformatics to analyze the physicochemical properties of *ANS* genes, secondary structure, promoter cis-acting elements, gene structure, evolutionary relationships, motif and expression levels of different tissues. In addition, the changes in peel anthocyanins content during different periods were analyzed by real-time PCR. These results provide new insights for further studies of the grape genome function and breeding.

## Result

### Identification and physicochemical properties analysis of grape ANS gene family

Using the amino acid sequence of the *Arabidopsis ANS* gene as the query sequence, a total of 121 genes were retrieved using the TBtools blast alignments and NCBI protein blast, and named *VvANS1*-*VvANS121* based on the location of the gene on the chromosome. The shortest amino acid length is 200aa (*VvANS96*) and the longest is 701aa (*VvANS52*). The molecular weight was between 22731.33 Da and 80177.52 Da. The isoelectric point of (PI) was in the range from 4.98 (*VvANS36*) to 9.32 (*VvANS110*), the PI value of *VvANS84* was 7, which was an neutral protein. In addition to the *VvANS66*, *VvANS76*, *VvANS80*, *VvANS96*, *VvANS110* and *VvANS114* were basic protein because their isoelectric point was greater than 7, all other were acidic protein. The instability index of 65 (53.72%) VvANS protein was greater than 40, indicating that these proteins were unstable proteins. Except for *VvANS76*, all other were hydrophilic proteins (Supplementary Table S1). Based on the physicochemical properties of proteins, predicted family members may play different functions.

### Evolutionary tree, motif, gene structure, domain analysis

**Fig. 1:**
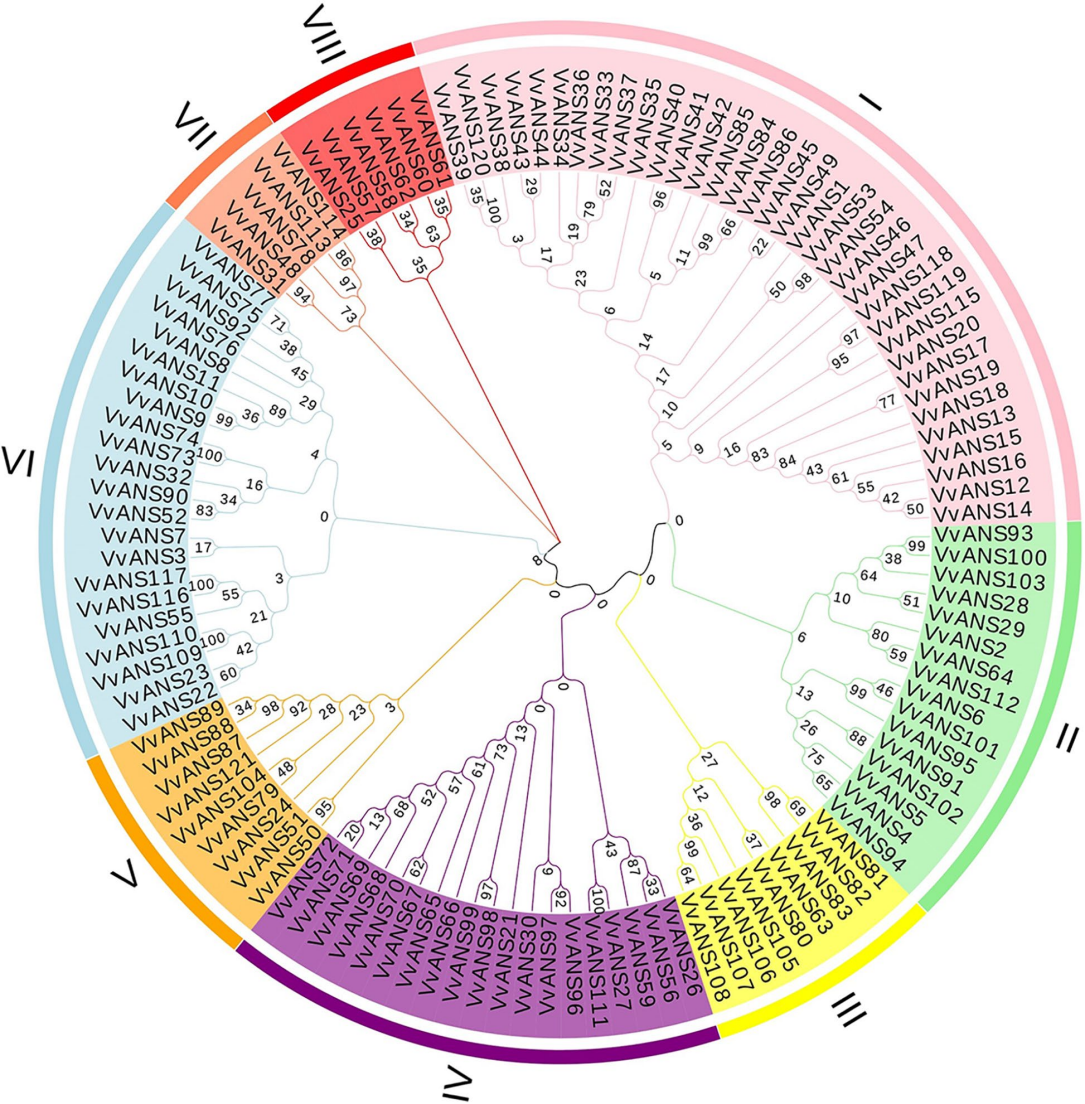
Phylogenetic analysis of the grape ANS gene family. Phylogenetic trees were constructed using the ANS protein sequences. Neighbor-joining method was adopted, and the bootstrap value was set to be equal to 1000. The similarity is calculated mainly through the progressive comparison method of sequence comparison

**Fig. 2:**
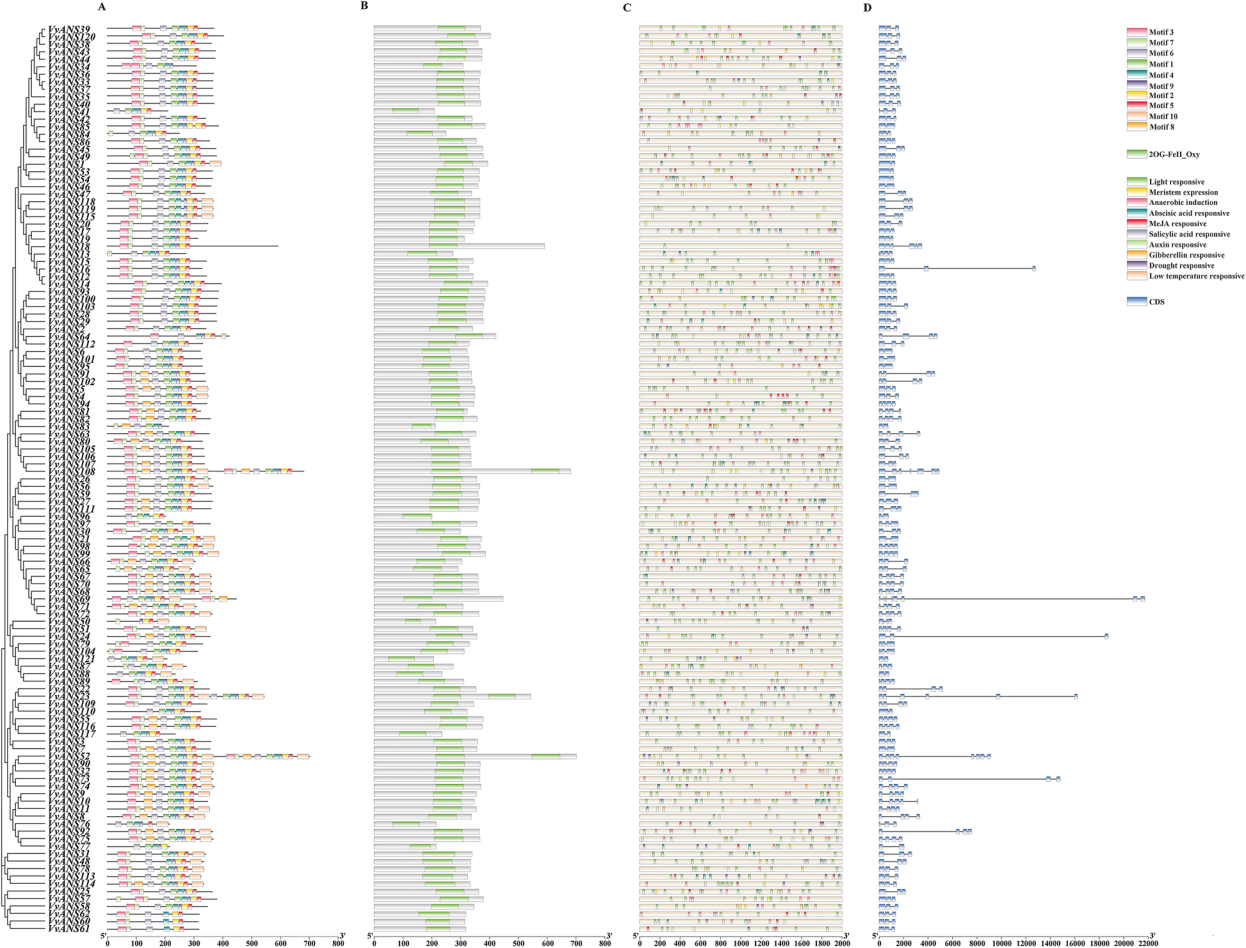
Analysis of motifs, domains, promoter cis-acting elements and gene structure of *VvANS* gene. **A** Analysis of conserved motif of *VvANS* gene. **B** Analysis of conserved domain of *VvANS* gene. **C** Analysis of cis-acting elements of *VvANS* gene promoter. **D** The exon–intron structure of *VvANS* genes

The ANS amino acid sequences of grape were used to construct a phylogenetic tree (Fig. 1), they were divided into 8 subgroups based on evolutionary relationships, with I subgroup having the most genes and VII subgroup having the least genes. *VvANS* genes contained 2–8 exons, most genes contain 3 exons, 19 genes contain only 2 exons, and *VvANS69* and *VvANS52* contain 8 exons, and the gene structure in the same branch genes has the same distribution and length (Fig. 2). The conserved motif of VvANS gene family proteins is predicted from the MEME website (Fig. 2), with a total of 10 motifs, the N terminus of most of the sequences are motif3, the C terminus is motif5. 43 genes contain motif10, 40 genes contain motif2, *VvANS23*, *VvANS52* and *VvANS108* all contain the repetitive motif, and genes in the same clade have similar motif positions and numbers. In the NCBI-CDD prediction, all *VvANS* genes had 2OG-Fe_Oxy (Fig. 2).

### Analysis of promoter cis-acting elements and tissue expression patterns

**Fig. 3:**
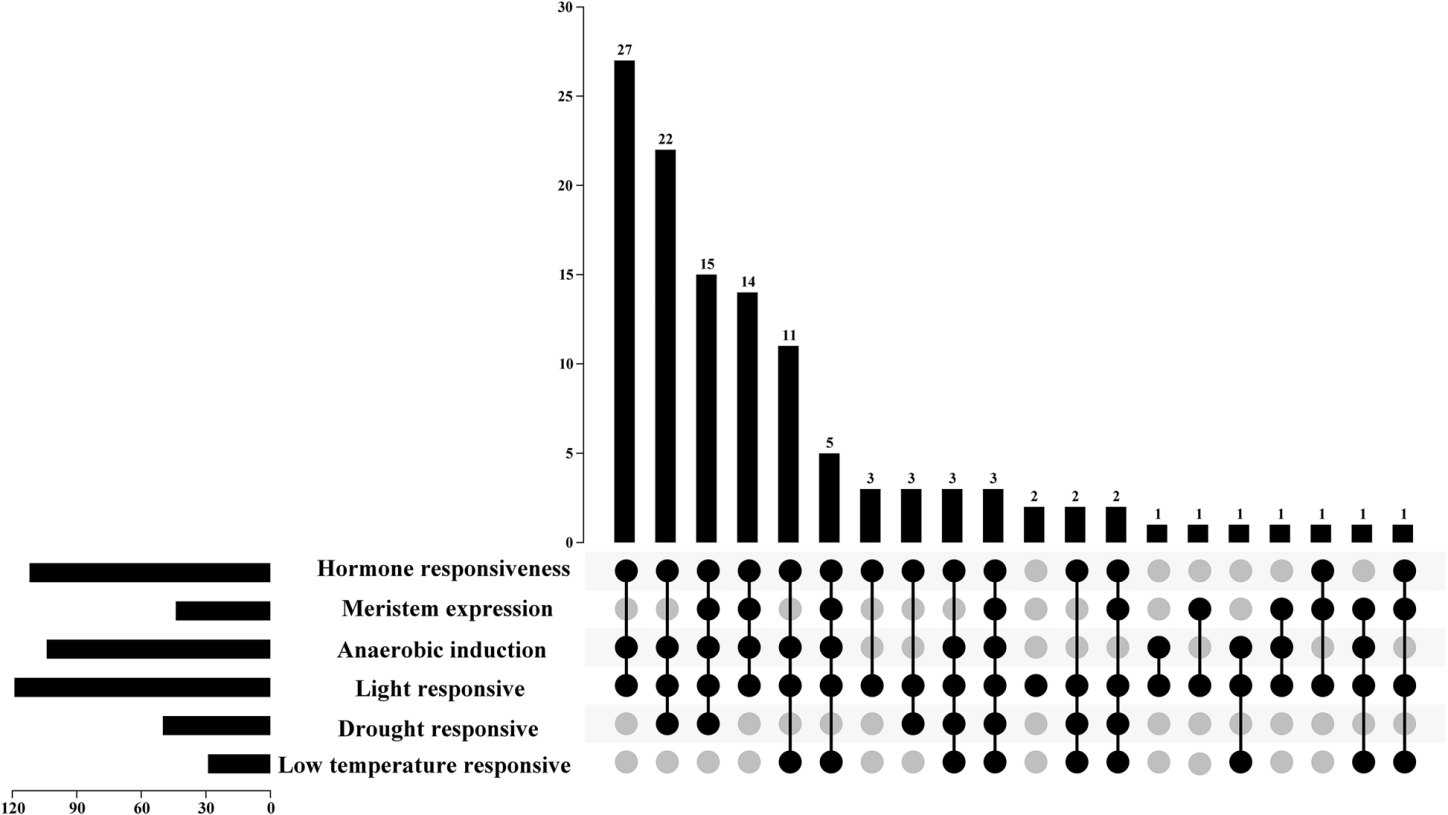
The intersection of promoter cis-acting elements. Numbers represent the number of involved genes

Cis-acting elements directly affect the function of downstream genes, and the cis-acting element analysis of the first 2000 bp of grape *ANS* genes. The results showed that *VvANS19* and *VvANS118* did not contain any acting elements, other VvANS genes mainly contained light, hormone, abiotic stress, meristem response elements. Hormone response elements contained auxin, gibberellin, abscisic acid, salicylic acid, methyl jasmonate response elements, and abiotic stress response elements contained low temperature, drought and anaerobic induction response elements (Fig. 2). Through the intersection of 119 genes between acting elements revealed that all 119 genes contained light response elements, including two genes containing only light response elements, namely *VvANS18* and *VvANS82*, and only three genes contained light, hormone, abiotic stress, meristem response elements, namely *VvANS24*, *VvANS88*, *VvANS114. VvANS79* only contains light response and anaerobic induction response elements, and *VvANS20* only contains light response and meristem response elements (Fig. 3).

**Fig. 4:**
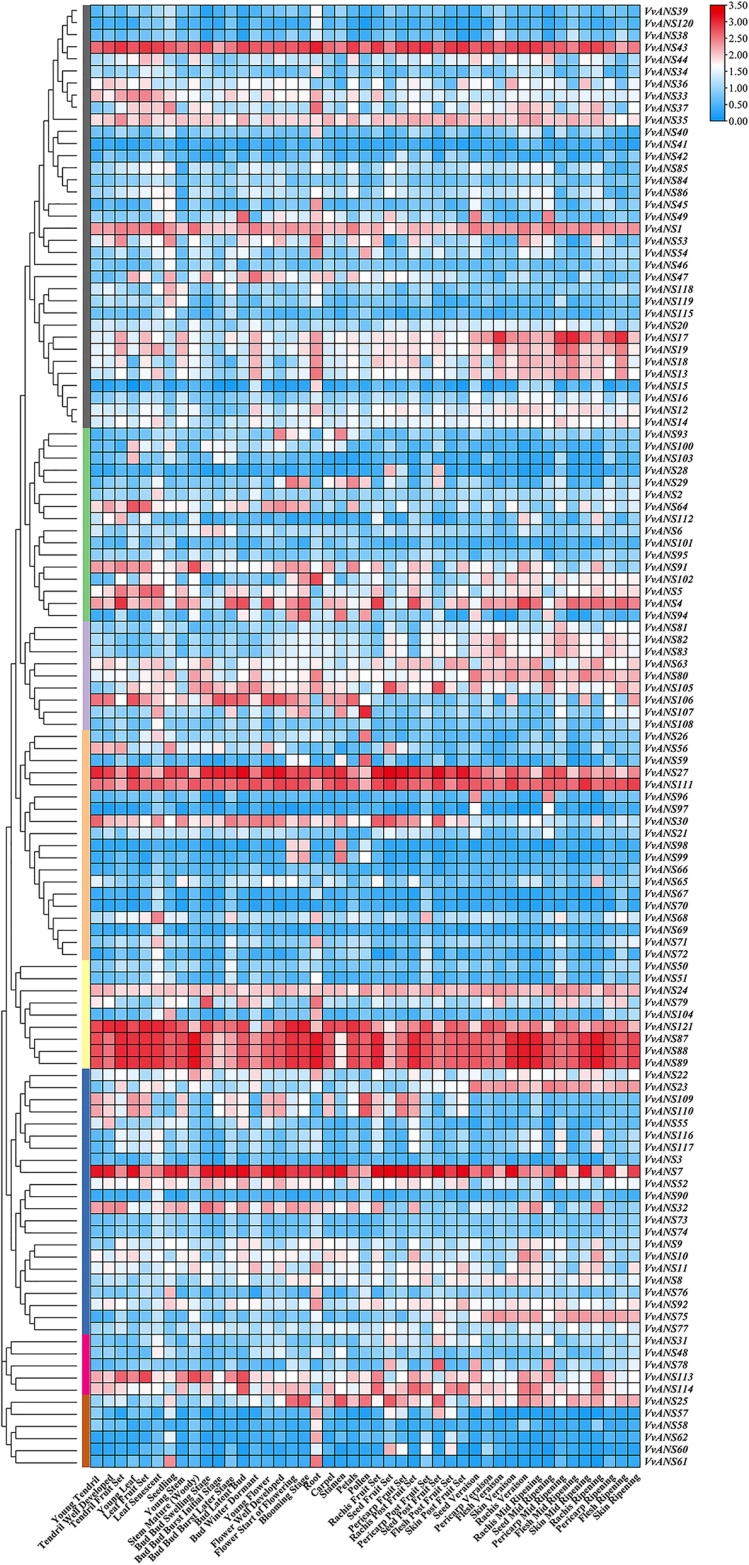
Expression of *ANS* gene in different tissues of grape. Red or blue shading represented the up-regulated or down-regulated expression level, respectively. The scale denoted the relative expression level

Analysis of the expression levels of *VvANS* genes in different tissues (Fig. 4), showed that genes in the same subfamily with similar expression levels. *VvANS1*, *VvANS7*, *VvANS27*, *VvANS43*, *VvANS87*, *VvANS88*, *VvANS89*, *VvANS111* and *VvANS121* were significantly upregulated in all tissues. *VvANS1* and *VvANS43* are located in subgroupI, *VvANS27* and *VvANS111* are located in subgroupIV, *VvANS87*, *VvANS88*, *VvANS89* and *VvANS121* are located in subgroupV, *VvANS7* is located in the subgroupVI. The expression of *VvANS105* was higher in seeds during and after fruit setting. *VvANS60* was higher in seeds during the fruiting period and lower in other periods, and *VvANS17* was higher in flesh and mid-mature peel. *VvANS49* was higher in seeds during mid-maturity, and *VvANS23* and *VvANS75* were higher in seeds, peel and flesh during mid-maturity and maturity, indicating that these genes may be involved in pigment synthesis.

### Chromosomal localization and collinearity analysis

**Fig. 5:**
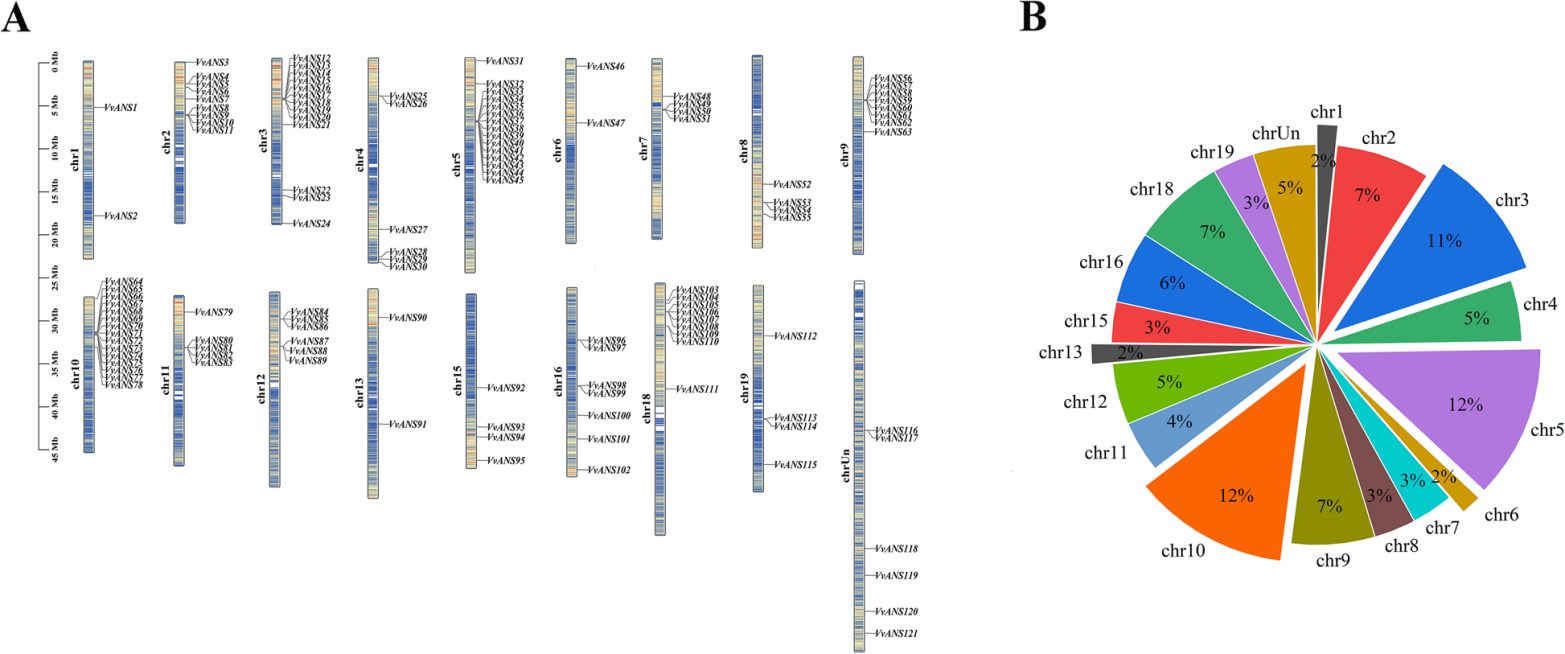
Chromosomal localization and distribution of the VvANS gene family. **A** Chromosomal localization of the VvANS gene family. The left scale indicates the chromosome length (Mb), with *ANS* gene markers on the right side of each chromosome. Different chromosomal colors indicate different gene densities, with red indicating the highest density and blue the lowest density. **B** Chromosome distribution of the VvANS gene family

Chromosome mapping analysis using TBtools software (Version 1.108) showed that a total of 121 genes were distributed on 18 chromosomes, of which 6 genes were located on unknown chromosomes. There are only 2 genes on chromosomes 1, 6 and 13. 9 genes on chromosome 2 and 18, 6 genes on chromosome 4 and 12. There are 15 genes on chromosomes 5 and 10, 4 genes on chromosomes 7, 8, 15, and 19. 13, 8, 5 and 7 genes on chromosome 3, 9, 11 and 16, respectively (Fig. 5A). Genes on chromosomes 5 and 10 are the most widely distributed, accounting for 12% of the total genes, followed by chromosomes 3 with 11% of the total genes, and genes on chromosomes 1, 6 and 13 each with 2% of the total genes (Fig. 5B).

**Fig. 6:**
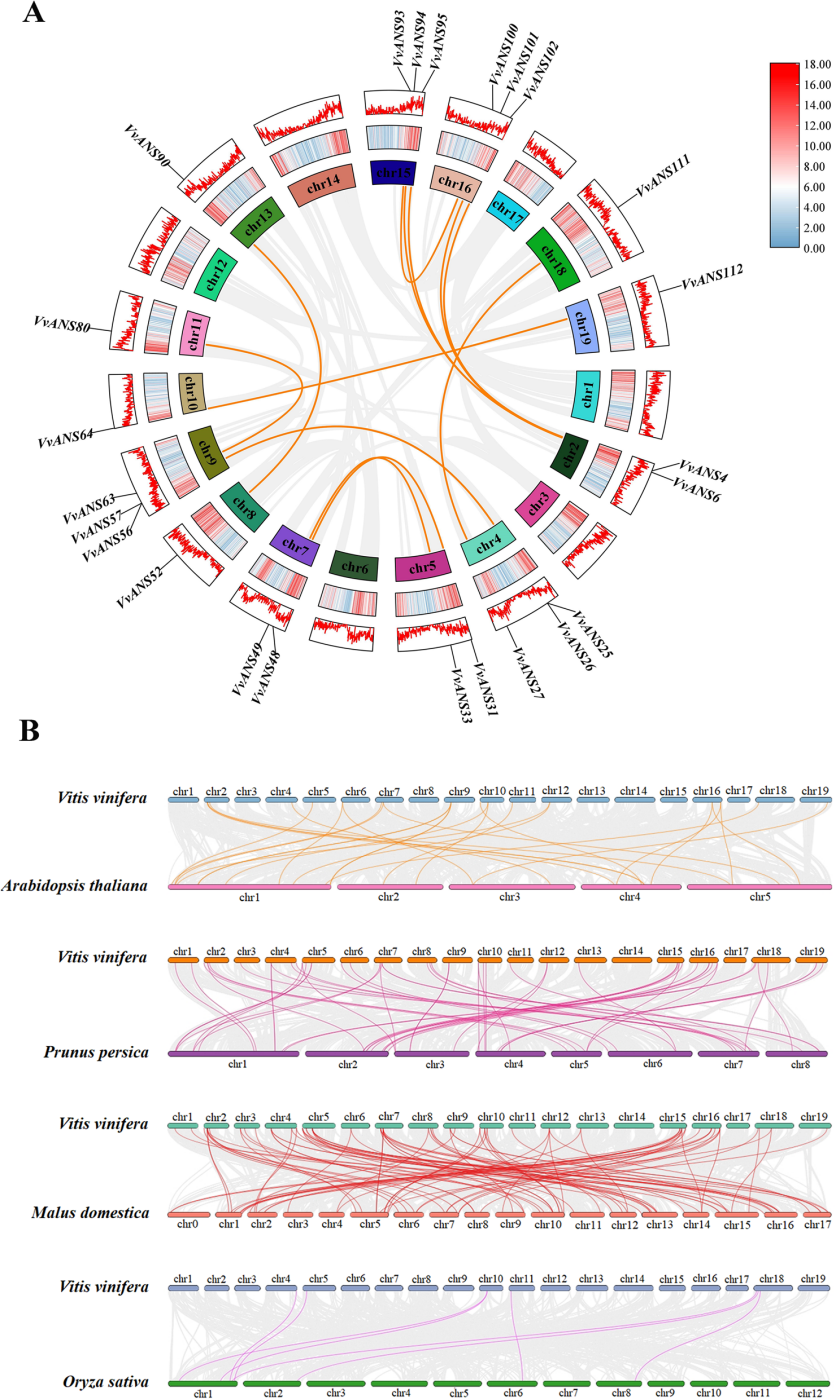
Collinearity analysis of VvANS gene families. **A** Collinearity analysis of *VvANS*. The gray lines represent all collinear blocks in the grape genome, and the orange lines represent gene pairs between the *VvANS* genes. **B** Collinearity analysis of *ANS* gene in grape and four representative plants. The gray lines in the background show collinearity between the grape and *Arabidopsis thaliana*, peach, apple, and rice genomes. The yellow lines show collinearity between the *VvANS* gene and *Arabidopsis thaliana*, the peach lines show collinearity between the *VvANS* gene and peach, and the red lines show collinearity between the *VvANS* gene and apple. The purple lines represent collinear gene pairs between the *VvANS* gene and rice

To further understand the evolutionary relationships of gene families, within-and inter-species collinearity analysis by the MCScanX tool of TBtools. A total of 13 collinearity relationships were found within the VvANS gene family species (Fig. 6A), located on chromosomes chr2, chr4, chr5, chr7, chr8, chr9, chr10, chr11, chr13, chr15, chr16, chr18, chr19, respectively. They are *VvANS4*/*VvANS94*, *VvANS4*/*VvANS102*, *VvANS6*/*VvANS95*, *VvANS6*/*VvANS101*, *VvANS57*/*VvANS25*, *VvANS56*/*VvANS26*, *VvANS80/VvANS63*, *VvANS90*/*VvANS52*, *VvANS49*/*VvANS33*, *VvANS48*/*VvANS31*, *VvANS64*/*VvANS112*, *VvANS27*/*VvANS111* and *VvANS93*/*VvANS100*. Among these, *VvANS4* and *VvANS6* both have two tandem repeats. These results suggest that some *VvANS* genes probably arise by gene duplication, which may have similar functions.

In order to further explore the evolutionary relationship of VvANS gene family, the collinearity map was drawn with four representative plants (Fig. 6B), with 27, 55, 93 and 8 pairs of *Arabidopsis*, peach, apple and rice, indicating that there are more homologous genes of grape and dicots than those of monocots.

### Codon preference and selection pressure analysis

**Fig. 7:**
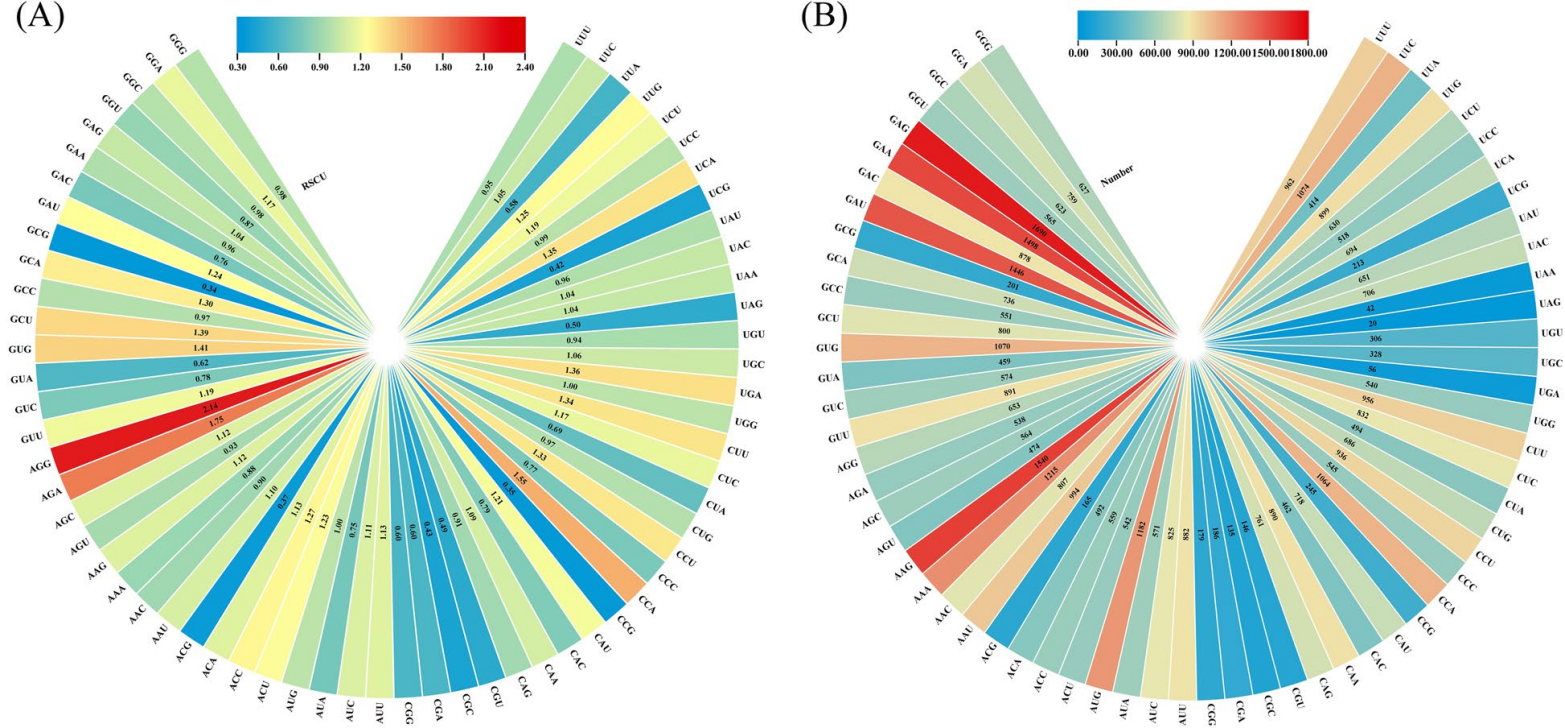
Relative synonymous codon usage and quantity analysis of *ANS* gene codon in grape. **A** Usage of synonymous codons. **B** The preferred number of synonymous codons

**Fig. 8:**
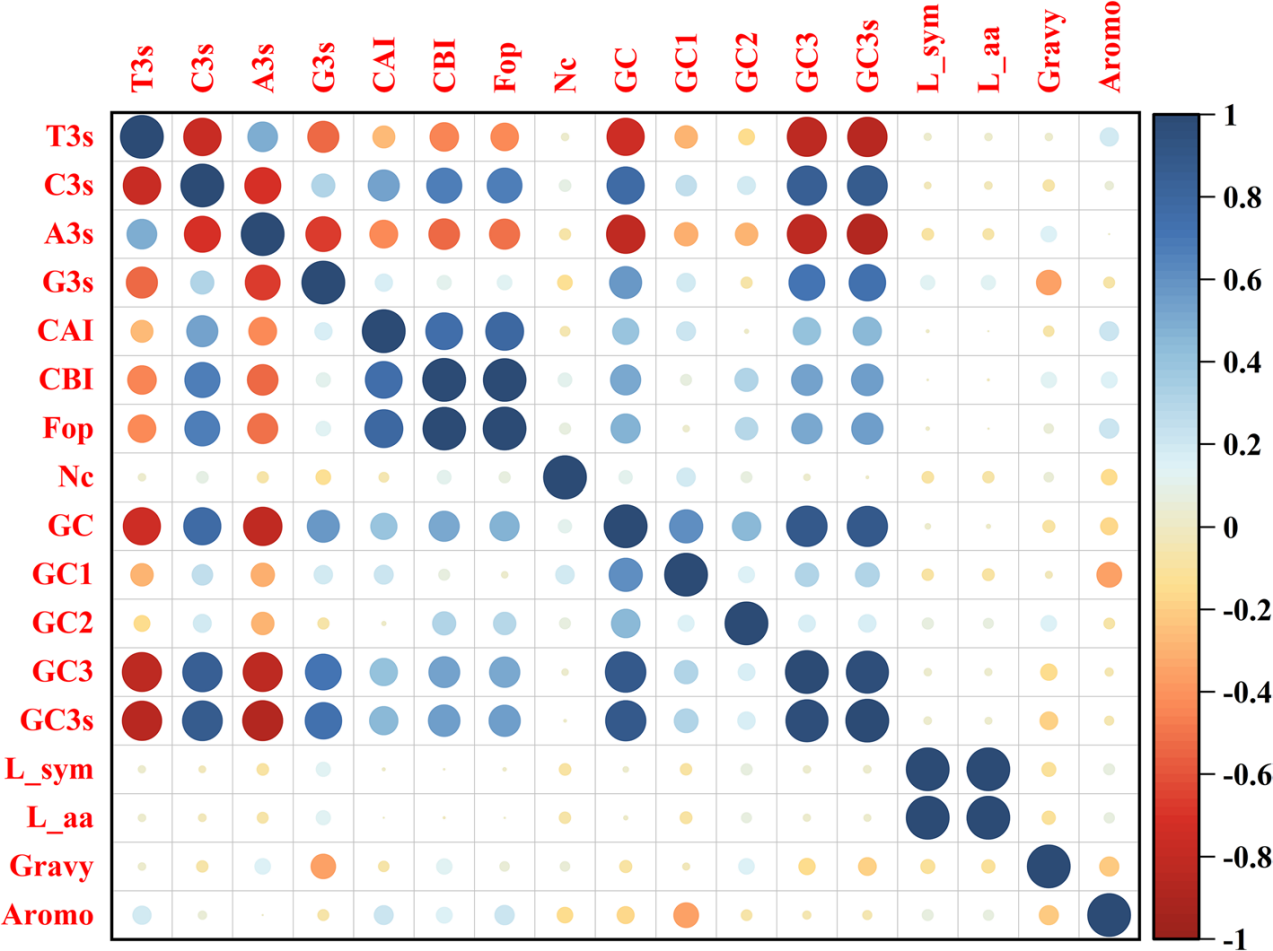
Correlation analysis of *VvANS* gene codon. Blue indicates positive correlation, red indicates negative correlation, and white indicates no correlation. The darker the color, the larger the circle and the stronger the correlation, and vice versa. The number of observations (n) of the correlation coefficient is 121

The components of the codons include CAI (codon adaptation index), CBI (codon bias index), Fop (frequency of optical codons), Nc (effective number of codon), GC (guanine and cytosine), GC1 (GC at the first codon position), GC2 (GC at the second codon position) and GC3 (GC at the third codon position), etc. Analyzed the frequency of relative synonymous codon usage in the grape genome, RSCU ≥ 1 was found with a total of 33 codons (Fig. 7A), namely, UUC、UUG、UCU、UCA、UAC、UAA、UGC、UGA、UGG、CUU、CUC、CCU、CCA、CAU、CAA、AUU、AUC、AUG、ACU、ACC、ACA、AAU、AAG、AGC、AGA、AGG、GUU、GUG、GCU、GCA、GAU、GAG、GGA, among 10 codons in the third position are U, 9 are A, 7 are C, 7 are G, this suggests that the third codon of the grape ANS protein prefers A or U. The codons in the third position were 8,795 (U), 5,271 (A), 4,888 (C), and 7,574 (G), representing 33.15%, 19.87%, 18.43%, and 28.55% of the total codons, respectively (Fig. 7 B). Among the grape *ANS* genes, the mean values of CAI, CBI, Nc and Fop were 0.202, -0.050, 0.384, and 54.30, respectively. The GC content of *VvANS* genes ranged from 39.82% to 53.55%, the content of GC1 ranged from 44.74% to 60.75%, the GC2 content ranged from 30.86% to 42.98%, the GC3 content ranged from 36.72% to 65.99%, and the average values of GC, GC1, GC2 and GC3 were 45.91%, 52.95%, 36.50% and 48.30%, respectively. The 12 genes were found to have Nc values less than 50, respectively *VvANS3*, *VvANS4*, *VvANS8*, *VvANS9*, *VvANS26*, *VvANS52*, *VvANS86*, *VvANS90*, *VvANS101*, *VvANS106*, *VvANS110* and *VvANS119*, indicating the strong codon preference of the 12 genes (Supplementary Table S2). Correlation analysis showed that T3s was positively correlated with A3s, CAI, CBI and Fop were all negatively correlated with T3s and A3s, Nc was negatively correlated with A3s, G3s and CAI, and was positively correlated with CBI and GC1, GC with C3s, G3s, CAI, CBI, Fop, GC1, GC2, GC3 and GC3s, but negatively correlated with T3s and A3s (Fig. 8).

**Fig. 9:**
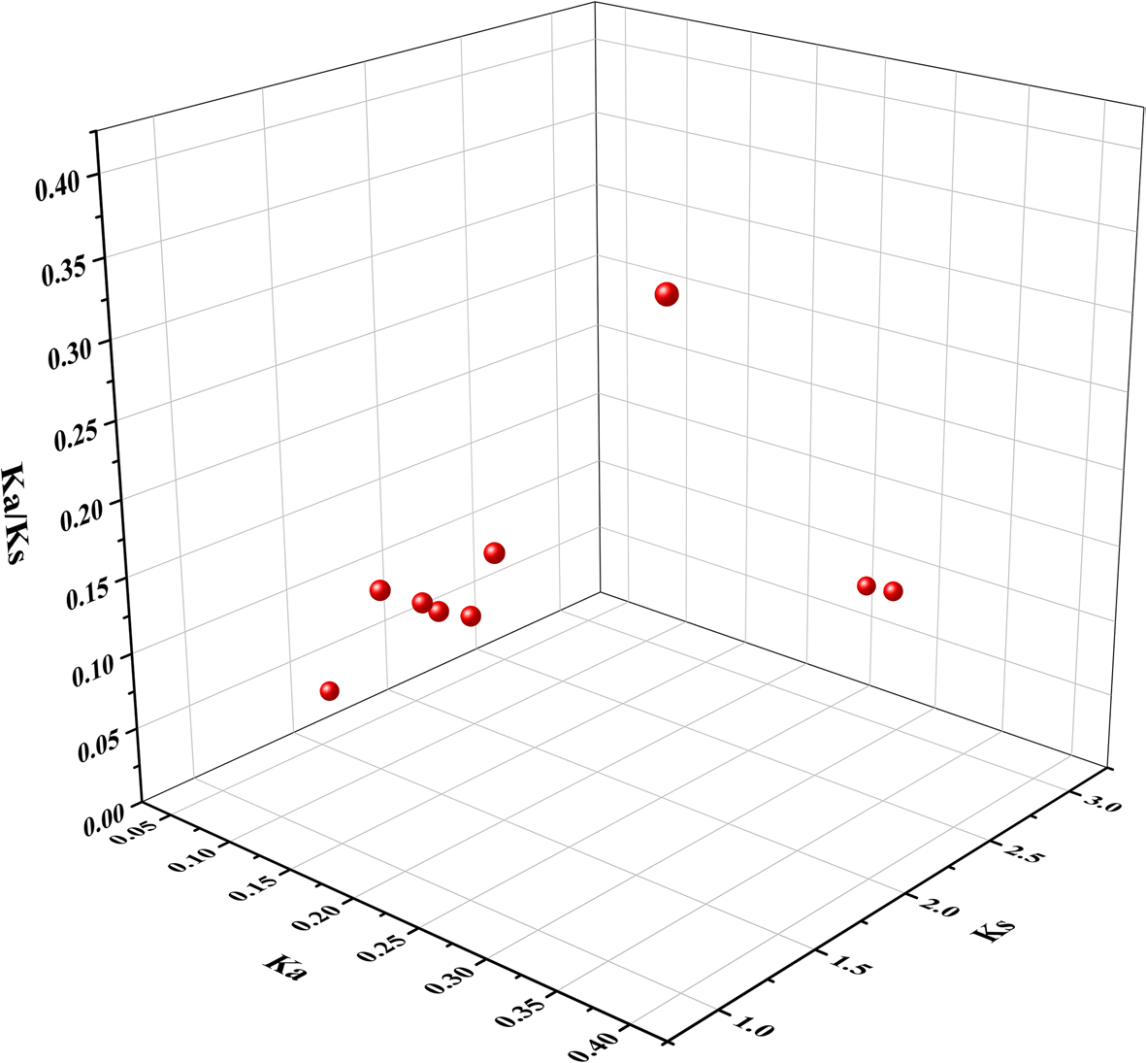
Selective pressure analysis of *VvANS* homologous gene pairs. The X axis represents the Ka value, the Y axis represents the Ks value, and the Z axis represents the ratio of Ka to Ks

The Ka/Ks allowed estimation of their evolutionary selection pressure to further understand the evolutionary relationships of the grape ANS gene family (Fig. 9). From 13 pairs of genes with collinear relationship, the Ka/Ks of 9 pairs of genes were calculated to be less than 1, suggesting that the grape ANS gene family may be dominated by purifying selection.

### Secondary structure, subcellular localization and protein interaction of ANS family proteins in grape

Secondary structure prediction showed that none of the proteins had β-angles, mainly α-helices, irregular coiled and extended chains, with the most irregular coiled (40.43% -59.20%), followed by α-helices (18.59%-45.33%) and the least extended chain (8.58%-26.52%) (Supplementary Table S3). According to the subcellular localization prediction, the members of the grape ANS gene family were mainly located in the chloroplast, cytoplasm and nucleus, and only 14 genes were located in the Golgi apparatus (Supplementary Table S3).

**Fig. 10:**
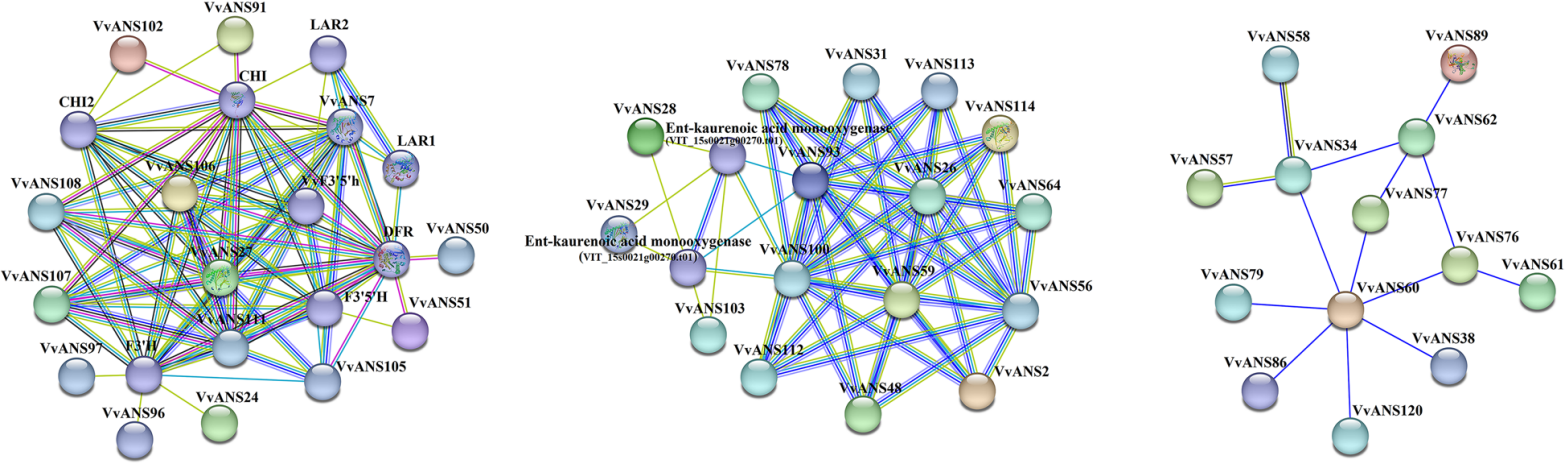
Analysis of protein interaction of ANS gene family in grape. Nodes indicate proteins. Empty nodes indicate the protein of unknown 3D structures, and filled nodes indicate that some 3D structures are known or predicted. The connection between nodes indicates the interaction between proteins, and different colors correspond to different types of interaction

The interactions between 121 VvANS proteins were predicted by the STRING online website (Fig. 10), and the results showed that 43 VvANS proteins may interact, and these 43 VvANS proteins interact to form three independent protein interaction networks (PPI networks). VvANS7, VvANS27, VvANS50, VvANS51, VvANS105, VvANS106, VvANS107, VvANS108 and VvANS111 all interact with DFR. VvANS24, VvANS96, VvANS97, VvANS105, VvANS106, VvANS107, VvANS108 and VvANS111 interact with F3 ′ H. VvANS7, VvANS27, VvANS91, VvANS102, VvANS106, VvANS107, VvANS108 and VvANS111 interact with CHI. VvANS7, VvANS27, VvANS91, VvANS102 and VvANS111 interact with CHI2. VvANS28, VvANS29, VvANS93, VvANS100 and VvANS103 interact with the grape protein VIT_15s0021g00270.t01.

### Determination of anthocyanin content and expression analysis of VvANS gene family in grape at different coloring stage

**Fig. 11:**
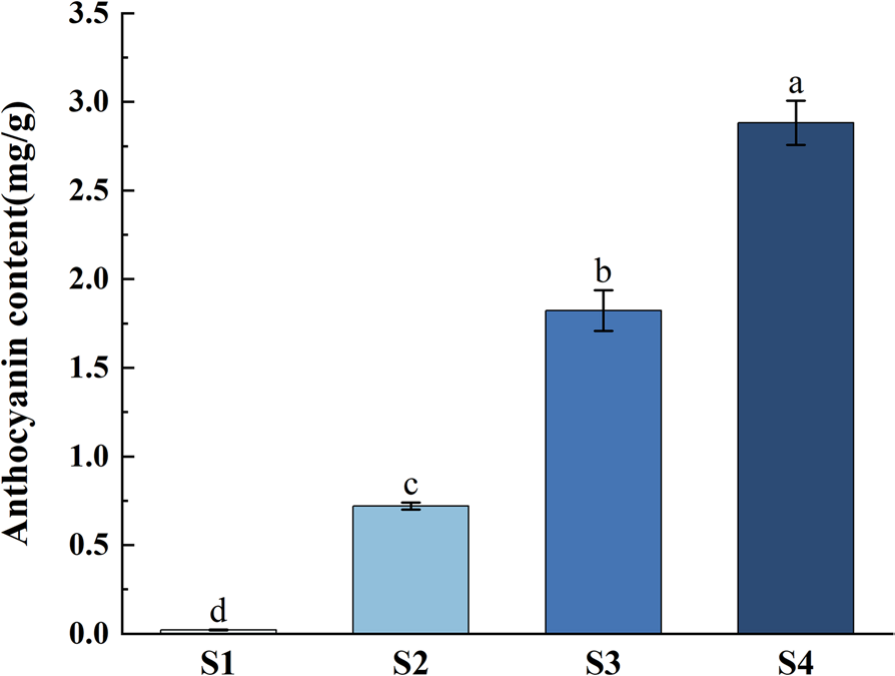
Content of grape anthocyanins in 4 periods. S1 represents the one week before color change, S2 represents the initial coloration period, S3 represents the 50% coloration, and S4 represents the complete coloration. The critical value is 4.07 by checking the F critical value table

**Fig. 12:**
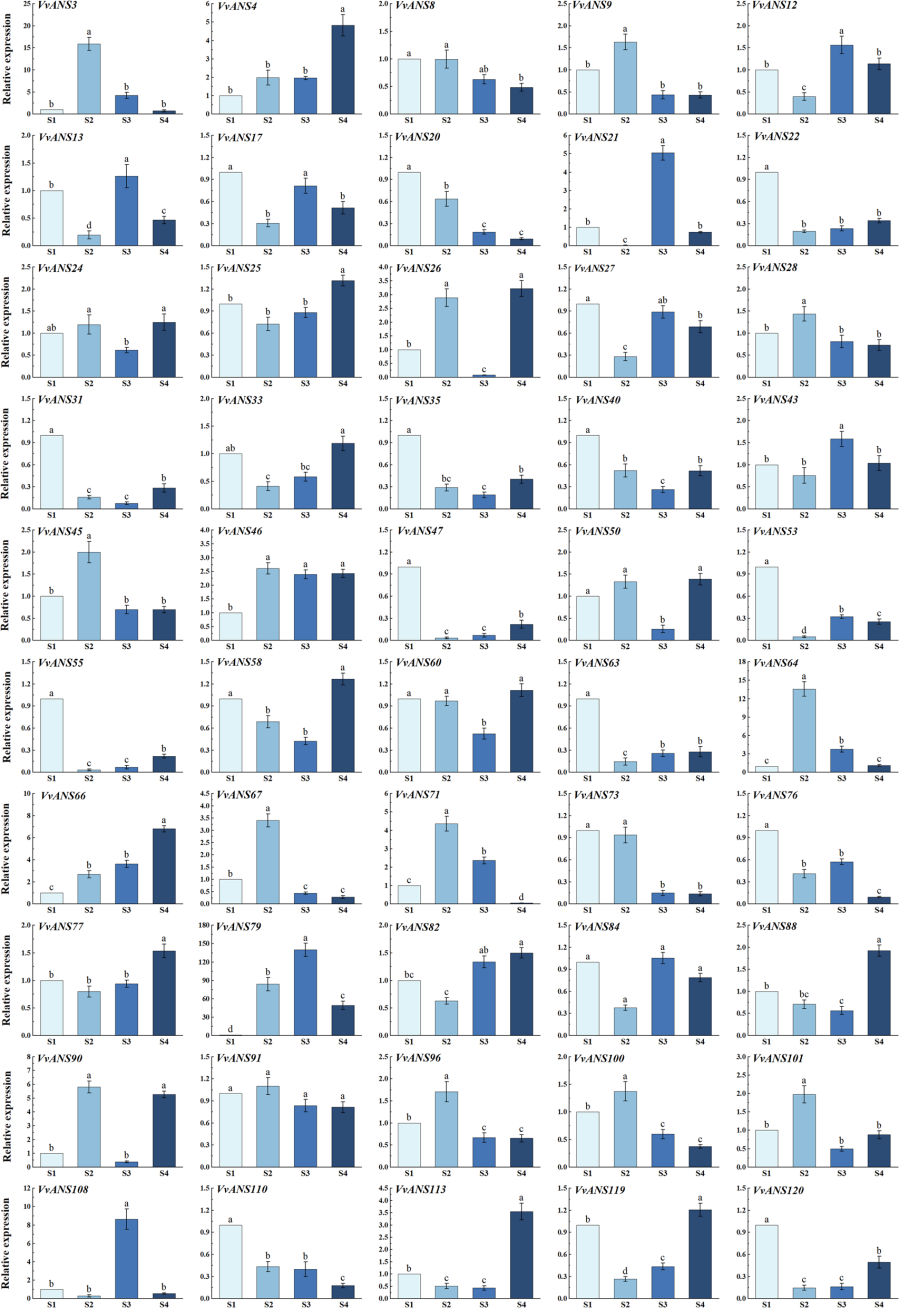
Relative expression levels of *ANS* gene in grape treated at different periods. S1 period was used as control. The 2^−∆∆Ct^ method was used to calculate the relative expression. Error bars represent the mean ± SE from three biological repeats. Different letters denote significant differences, whereas the same lowercase letters indicate no statistical difference (*P* < 0.05). The critical value of each gene was 4.07 by checking the F critical value table

From S1 to S4 are different coloring stages of grapes, which are one week before color change (S1), the initial coloration period (S2), 50% coloration (S3) and complete coloration (S4), respectively (Fig. 11). With the increase of fruit coloring, anthocyanin content gradually increases. qRT-PCR analysis showed that the expression of *VvANS* gene was found in all stages, indicating that the ANS gene family may be involved in all stages of grape color transformation, but the expression level varied irregularly in different growth stages (Fig. 12).

*VvANS17*, *VvANS20*, *VvANS22*, *VvANS27*, *VvANS31*, *VvANS35*, *VvANS40*, *VvANS47*, *VvANS53*, *VvANS55*, *VvANS63*, *VvANS73*, *VvANS76*, *VvANS110* and *VvANS120* had the highest expression in S1 period. *VvANS3*, *VvANS9*, *VvANS28*, VvANS45, *VvANS64*, *VvANS67*, *VvANS71*, *VvANS90*, *VvANS96*, *VvANS100* and *VvANS101* had the highest expression in S2 period. The expression of *VvANS64* in S2 period was 13.6 times that in S1 period. The expression of *VvANS3* in S2 was 8.6 times higher than that in S1. The expression of *VvANS90* in S2 was 5.8 times that in S1. *VvANS12*, *VvANS13*, *VvANS21*, *VvANS43*, *VvANS79* and *VvANS108* had the highest expression in S3 period. The expression of *VvANS79* in S3 was 139 times that in S1. The expression of *VvANS108* in S3 was 8.6 times that in S1. *VvANS21* expression in S3 was 5 times higher than that in S1. *VvANS4*, *VvANS24*, *VvANS25*, *VvANS26*, *VvANS33*, *VvANS58*, *VvANS66*, *VvANS77*, *VvANS82*, *VvANS88*, *VvANS113* and *VvANS119* had the highest expression levels in S4 period. *VvANS66* expression in S4 period was 6.8 times that in S1 period. The expression of *VvANS4* in S4 was 4.8 times that in S1. The expression level of *VvANS113* in S4 was 3.5 times that in S1.

## Discussion

Anthocyanin synthetase is a key enzyme in plant anthocyanin bioanabolic pathway, which can catalyze the conversion of colorless anthocyanins to colored anthocyanins, and plays an important role in plant color formation [26]. Menssen [27] cloned and identified the first *LDOX* gene from maize A2 mutant using transposon labeling technology. *ANS* genes have been studied and cloned in many crops, such as *Arabidopsis*, apple, cacao, *Ginkgo biloba*, etc. [28–31], however, *ANS* genes involved in anthocyanins synthesis have rarely been studied in grapes. In this study, we found 121 grape *ANS* genes, which is a large gene family. Physicochemical properties analysis showed that most of the *VvANS* genes were acidic proteins, while only 6 were basic proteins and 1 was neutral protein (Supplementary Table S1). Each *VvANS* gene contained the conserved domain 2OG-Fe_Oxy (Fig. 2), which is consistent with that studied in *Punica granatum* [32]. Subcellular localization found that *VvANS* gene was mainly localized in the cytoplasm, chloroplast and nucleus (Supplementary Table S3), which is consistent with the results studied in Cabernet Sauvignon grape [33]. 13 collinearity pairs in the VvANS gene family, namely *VvANS4*/*VvANS94*, *VvANS4*/*VvANS102*, *VvANS6*/*VvANS95*, *VvANS6*/*VvANS101*, *VvANS57*/*VvANS25*, *VvANS56*/*VvANS26*, VvANS80/VvANS63, *VvANS90*/*VvANS52*, *VvANS49*/*VvANS33*, *VvANS48*/*VvANS31*, *VvANS64/VvANS112*, *VvANS27*/*VvANS111* and *VvANS93*/*VvANS100.* Both *VvANS4* and *VvANS6* have two tandem repeats, these genes with colinearity are all on close branches of the same subfamily. This suggests that these genes with colinear relationships may have similar functions.

The promoter of a gene may determine the function of a gene. In this study, cis-acting element analysis of the first 2000 bp sequence of *VvANS* gene found that there were more elements responsive to light, hormone and abiotic stress. Under low light conditions, *Dendrobium* color becomes lighter, mainly because *DsDFR* and *DsANS* expression in *Dendrobium* are light dependent, low light can down-regulate *DsDFR* and *DsANS* expression and reduce anthocyanin accumulation [34]. The up-regulated expression of structural genes (*PAL*, *CHS*, *CHI*, *F3H*, *F3'H*, *FLS*, *DFR*, *ANS*, *UFGT*) and regulatory genes (*McMYB10*) that can promote anthocyanin synthesis can promote the pigmentation and accumulation of anthocyanin in leaves and callus of begonia under long sunshine conditions [35]. ABA accelerates the development of fruit color by activating *PAL*, *CHS* and *ANS*, key genes in the phenylc/flavonoid and anthocyanin pathways [36]. In addition, a number of ion transporters as well as cellular signaling pathways associated with the stress response are induced by ABA, flavonoid and anthocyanin pathways [37, 38].

By protein interaction analysis, 43 interaction between the proteins and the DFR, F3′H, CHI, CHI2 and the grape protein VIT_15s0021g00270.t01. Dihydroflavonol 4-reductase (DFR) is a key enzyme in the anthocyanin biosynthesis pathway, and play an important role in plant and fruit coloration [39]. Two genes (*GlaDFR1* and *GlaDFR2*) cloned from gentian were overexpressed into tobacco plants, and the petals were found to be darker than the wild type in the T1 generation [40]. Flavonoid 3′ -hydroxylase (F3′H) is the first key enzyme in the anthocyanin synthesis pathway from dihydrokanafalcohol, which plays an important role in regulating flower color and fruit color. *Brassica napus* and *Petunia hybrida* pollinate coloured flowers and *Arabidopsis* self-pollinate white flowers, so F3′H expression is very high in rapeseed and petunia organs [41]. Charcone isomerase (CHI) is a key and rate-limiting enzyme in the synthesis of anthocyanins. This enzyme (CHI) and chalcone synthase (CHS) cooperate to regulate the synthesis of anthocyanins. CHI participates in the isomerization of chalcone and catalyzes the generation of chalcone to naringenin [42].

Studies have shown that the distribution of flavonoids and anthocyanins in different tissues and organs of plants is specific [43]. In peach, it was found that the expression of *PpANS* gene was higher in fruit skin, fruit flesh and flower, and the expression level was the highest in peach skin [44]. *FcANS1* transcripts were only expressed in root tips, terminal buds, young leaves and young stems of fig tree, but not in mature leaves, stems or petioles [45]. Jiang [46] found in the study of eggplant that the expression of *SmANS* gene was the highest in the peel and the second in the petal. In this study, on the basis of the molecular analysis, analyzed the expression of *VvANS* genes in different tissues, only a few genes showed higher expression in all tissues, expression of some genes is tissue-specific, the expression of *VvANS105* was higher in seeds during and after fruit setting. *VvANS60* was higher in seeds during the fruiting period and lower in other periods, and *VvANS17* was higher in flesh and mid-mature peel. *VvANS49* was high in seeds during mid-maturity, and *VvANS23* and *VvANS75* in seeds, peel, and flesh during mid-maturity and maturity. These results showed that the expression of *ANS* gene was higher in the parts with higher anthocyanin content.

A large number of studies have found that the expression of *ANS* has a certain correlation with the content of anthocyanins. Previous studies in Zoysiagrass (*Zoysia japonica* Steud.) showed that *ZjANS* gene expression was up-regulated in purple spike and stolons, while *ZjANS* gene expression was lower in green varieties [47]. The insertion mutation of 5 bp in the coding region of *ANS* gene in raspberry (*Rubus idaeus*) leads to the premature termination of amino acid translation, which leads to the reduction of pigment in raspberry fruit [48]. Carbone [49] found that the expression level of *LDOX* gene was low in the strawberry fruit turning red stage, and then showed an increasing trend. Shi [50] found that the expression level of *LDOX* genes in red petals of *Magnolia* was significantly higher than that in white petals. Boss [51] studied the expression of *LDOX* gene in the peel of eight different grape varieties, and found that the expression of *LDOX* gene was higher in the peel of red varieties, while the relative expression of *LDOX* gene was lower in the peel of white varieties. The qRT-PCR analysis in this study showed that *VvANS17*, *VvANS20*, *VvANS22*, *VvANS27*, *VvANS31*, *VvANS35*, *VvANS40*, *VvANS47*, VvANS53, *VvANS55*, *VvANS63*, *VvANS73*, *VvANS76*, *VvANS110* and *VvANS120.* The expression of these genes was highest in the S1 period, indicating that these genes may be involved in transcriptional regulation before grape coloring. This was similar to that found in mangoes, where the expression of *ANS* gene was highest in green peels, followed by in red peels, and lowest in yellow peels [52]. *VvANS3*, *VvANS9*, *VvANS28*, *VvANS45*, *VvANS64*, *VvANS67*, *VvANS71*, *VvANS90*, *VvANS96*, *VvANS100* and *VvANS101* had the highest expression in S2 period. *VvANS12*, *VvANS13*, *VvANS21*, *VvANS43*, *VvANS79* and *VvANS108* have the highest expression levels in S3 period, indicating that these genes play an important role in the fruit coloring process, which is consistent with the above research results. With the deepening of pigment, the expression level of *ANS* gene is gradually up-regulated. *VvANS4*, *VvANS24*, *VvANS25*, *VvANS26*, *VvANS33*, *VvANS58*, *VvANS66*, *VvANS77*, *VvANS82*, Vv*A*NS88, *VvANS113* and *VvANS119* had the highest expression in S4 period. These genes may be involved in the modification and degradation of anthocyanins at the late stage of anthocyanin synthesis.

## Conclusion

In this study, 121 *VvANS* genes were found and distributed in 18 chromosomes, which could be divided into 8 subfamilies according to the evolutionary relationship. The analysis of promoter cis-acting elements showed that *VvANS* gene contains many response elements related to anthocyanin synthesis, such as light elements, auxin, gibberellin, abscisic acid, salicylic acid, methyl jasmonate response elements. Protein interaction prediction showed that some of the *VvANS* genes interact with the structural genes *DFR*, *CHI* and *F3'H* genes in the anthocyanin synthesis pathway. Fluorescence quantitative results showed that *VvANS4*, *VvANS46*, *VvANS55*, *VvANS66*, *VvANS79* and *VvANS108* were highly expressed in the green fruit stage, 50% coloring stage and full maturity stage, respectively. These genes can be used as candidate genes for subsequent functional studies. This study will provide a way to further understand the role of ANS gene family in grape development and coloration.

## Materials and methods

### Plant materials and treatments

Using 'Pinot noir' grape berries as research materials, the berries were collected from four periods of time: one week before color change, the initial coloration period, 50% coloration and complete coloration were rapidly peeled, accurately weighed and then quickly frozen with liquid nitrogen and stored at -80℃ until needed for further analysis.

### Identification of ANS genes family in grape

The amino acid sequences of *Arabidopsis ANS* gene were downloaded, from the TAIR database (*Arabidopsis thaliana* Information Resource, https://www.arabidopsis.org/). Grape genome and annotation information were downloaded from the phytozome v13 (https://phytozome.jgi.doe.gov/pz/portal.htm) [53]. Their amino acid sequences were utilized for homology comparison and removal of redundant sequences at TBtools [54]. The screening results were then combined with the 2OG-Fe_Oxy functional domain. We searched a total of 121 grape *ANS* genes and downloaded their gene length, CDS (coding sequence length) and amino acid sequences.

### Analysis of the physicochemical properties of the grape ANS gene family

The molecular weight (MW), isoelectric point (PI), instability coefficient, fat index, and hydrophilicity of the grape ANS family were analyzed from the online software ExPASy (https://web.expasy.org/protparam/) [55].

### Evolutionary tree, motif, gene structure, domain analysis

The multiple sequence alignment of the VvANS proteins was conducted using the ClustalX 1.83 software, and MEGA 7.0 software was used to construct phylogenetic trees with the bootstrap value set to 1000, and beautify was performed at the EVOLVIEW website (https://evolgenius.info//evolview-v2/#login) [56]. Gene structure prediction was constructed using TBtools software. The conserved motifs of proteins were constructed by the MEME (http://meme-suite.org/tools/meme) [57], the number of motifs was set to 10 and conserved domains of the proteins were analyzed at the NCBI-CDD site (https://www.ncbi.nlm.nih.gov/cdd/).

### Analysis of promoter cis-acting elements and tissue expression patterns

The 2000 bp upstream sequence of the *VvANS* gene initiation codon (ATG) was obtained using the TBtools software with the online software PlantCARE (http://bioinformatics.psb.ugent.be/webtools/plantcare/html/) [58] and plotted at TBtools (Version 1.108). The expression levels of grape *ANS* genes in different tissues were searched from the BAR database (https://bar.utoronto.ca/), including tendril, roots, stems, leaves, flowers, seeds, pericarp etc., were searched, and the selected data were log10- transformed at TBtools (Version 1.108).

### The ANS gene location and synteny analysis

The predicted of the position of the grape *ANS* gene on the chromosome was constructed from TBtools (Version 1.108) and was plotted at TBtools. To analyze the collinearity relationships of *VvANS* genes, the genome and annotation files of *Arabidopsis*, peach, apple, and rice used for collinearity analysis were downloaded from phytozome v13 (https://phytozome.jgi.doe.gov/pz/portal.html), and the gene pairs of the *ANS* genes were determined using TBtools synteny, and the diagram was drawn via TBtools (Version 1.108).

### Codon usage bias analysis

The codon usage characteristics of the CDS sequence of *VvANS* genes were analyzed using the online software CodonW 1.4.2 (http://codonw.sourceforge.net), including relative synonymous codon usage (RSCU), effective codon (ENC), codon bias index (CBI), codon adaptation index (CAI), optimal codon usage frequency (Fop), T3s, C3s, A3s, G3s. At EMBOSS (https://www.bioinformatics.nl/emboss-explorer/) were performed for calculation of the total GC content, GC1, GC2 and GC3 content, with the following data of T3s, C3s, A3s, G3s, CAI, CBI, Nc, Fop, GC, GC1, GC2, GC3, GC3s, L_sym, L_aa, GRAVY and Aromo Parameters for correlation analysis.

### Secondary structure, subcellular localization and protein interaction of ANS family proteins in grape

The NPS@: SOPMA website (https://npsa-prabi.ibcp.fr/cgi-bin/npsa_automat.pl?page=npsa_sopma.html) was used to predict the secondary structures of VvANS proteins. The online software WoLF PSORT (https://wolfpsort.hgc.jp/) was used to predict the subcellular localization of the VvANS proteins [59]. Protein–protein interaction network was performed via the STRING Version 11(https://string-db.org/) [60].

### RNA extraction and qRT-PCR analysis

The primers (Supplementary Table S4) were synthesized by Shanghai (Shanghai) Biological Engineering Co., Ltd. RNA was extracted from pinot noir fruit, reverse transcribed as single-stranded cDNA as template, and the quantitative reaction system was 20 μL: 2 μL cDNA, 1 μL each of upstream and downstream primers (10 μmol/L), 10 μL SYBR enzyme, 6 μL ddH_2_O. The reaction procedure was: 95℃ predenaturation for 30 s; 95℃ denaturation for 10 s, 60℃ annealing for 30 s, 72℃ extension for 30 s, 40 cycles; the test was repeated 3 times. Then the reaction procedure and the melting curve and the fluorescence value change curve were analyzed.

### Determination of anthocyanin content in grape peel during different developmental periods

The 1.0 g fruit was accurately weighed, ground in liquid nitrogen, put into a 10 mL centrifuge tube, rinsing the mortar with 1% HCl-methanol solution, and transferred to the test tube. The volume was fixed to the scale, and then mixed. Extraction was carried out at 4 °C for 20 min in the dark, during which the extraction was shaken several times. Samples were then filtered through 0.2 μm PES filters (Krackeler Scientific, Inc., Albany, NY, USA) and analyzed using TU-1900 double beam UV–visible spectrophotometer (Beijing Purkinje General Instrument Co. LTD). The solution was zeroed with 1% HCl-methanol solution as blank reference, and the absorbance of the solution was determined with filtrate at 600 nm and 530 nm, respectively, and repeated three times. Anthocyanin content (U) was expressed by the difference of absorbance value at wavelength 530 nm and 600 nm per gram of fresh weight peel tissue, i.e. U = (OD_530_-OD_600_)/gFW.

### Statistical analysis of the data

Statistical data were analyzed by Excel software, and the data were calculated and collated. After normalization of the data from three independent experiments, three repeated qRT-PCR quantitative data and anthocyanin content data were analyzed by Duncan method with One-way ANOVA in SPSS 22.0. *P* < 0.05 was significant difference and drew with Origin 2021. The experimental data were processed by the 2^−△△CT^ method [61].

### Supplementary Information

**Additional file 1: Supplementary** **Table S1.** Analysis of physicochemical properties of *VvANS* genes.

**Additional file 2: Supplementary Table S2.** *VvANS* gene codon preference parameter.

**Additional file 3: Supplementary** **Table S3.** Analysis of secondary structure and subcellular localization of VvANS protein.

**Additional file 4: Supplementary Table S4.** qRT-PCR primers for expression on analysis of VvANS gene.

## Data Availability

All data generated or analysed during this study are included in supplementary information files.
