# Supplementary material for: Genome-wide identification of grape ANS gene family and expression analysis at different fruit coloration stages
Source: BMC Plant Biol. 2023 Dec 9;23:632. doi: 10.1186/s12870-023-04648-3 (PMC10709965; doi:10.1186/s12870-023-04648-3)
Supplement: Supplementary file 1 — Additional file 1: Supplementary Table S1. Analysis of physicochemical properties of VvANS genes. [file 12870_2023_4648_MOESM1_ESM.docx]

**Supplementary** **Table S1.** Analysis of physicochemical properties of *VvANS* genes

| Gene name | Accession number | Amino acid numbers(aa) | Molecular weight (Da) | PI | Instability index | Aliphatic index | hydropathicity |
| --- | --- | --- | --- | --- | --- | --- | --- |
| *VvANS1* | *VIT_201s0011g05650.1* | 393 | 43770.98 | 6.28 | 33.16 | 82.34 | -0.224 |
| *VvANS2* | *VIT_201s0010g01650.1* | 340 | 38485.74 | 5.31 | 46.34 | 77.44 | -0.334 |
| *VvANS3* | *VIT_202s0234g00010.1* | 357 | 40184.16 | 5.83 | 35.01 | 88.96 | -0.230 |
| *VvANS4* | *VIT_202s0025g02960.1* | 348 | 39571.01 | 5.79 | 40.10 | 82.82 | -0.409 |
| *VvANS5* | *VIT_202s0025g02970.1* | 348 | 39295.50 | 5.65 | 42.88 | 84.20 | -0.346 |
| *VvANS6* | *VIT_202s0025g03440.1* | 321 | 36335.46 | 4.99 | 48.42 | 82.83 | -0.229 |
| *VvANS7* | *VIT_202s0025g04720.1* | 355 | 40193.25 | 5.63 | 48.13 | 90.31 | -0.368 |
| *VvANS8* | *VIT_202s0012g00360.1* | 336 | 37934.82 | 5.62 | 33.65 | 88.18 | -0.274 |
| *VvANS9* | *VIT_202s0012g00390.1* | 353 | 39705.90 | 5.39 | 34.07 | 94.67 | -0.212 |
| *VvANS10* | *VIT_202s0012g00400.1* | 346 | 38803.75 | 5.28 | 38.50 | 91.79 | -0.188 |
| *VvANS11* | *VIT_202s0012g00450.1* | 353 | 40116.23 | 5.85 | 43.96 | 87.82 | -0.303 |
| *VvANS12* | *VIT_203s0063g01150.1* | 342 | 39217.40 | 5.67 | 30.32 | 81.49 | -0.520 |
| *VvANS13* | *VIT_203s0063g01160.1* | 271 | 31378.58 | 5.78 | 36.82 | 78.71 | -0.568 |
| *VvANS14* | *VIT_203s0063g01180.1* | 393 | 44514.34 | 5.68 | 34.45 | 82.54 | -0.464 |
| *VvANS15* | *VIT_203s0063g01210.1* | 342 | 39166.43 | 6.04 | 40.24 | 81.81 | -0.500 |
| *VvANS16* | *VIT_203s0063g01250.1* | 327 | 37715.92 | 5.16 | 34.60 | 85.81 | -0.434 |
| *VvANS17* | *VIT_203s0063g01290.1* | 342 | 39263.50 | 5.93 | 37.76 | 83.19 | -0.500 |
| *VvANS18* | *VIT_203s0063g01310.1* | 590 | 66856.49 | 6.31 | 43.82 | 85.73 | -0.334 |
| *VvANS19* | *VIT_203s0063g01330.1* | 312 | 35595.38 | 5.37 | 35.83 | 83.08 | -0.418 |
| *VvANS20* | *VIT_203s0063g01340.1* | 347 | 39738.26 | 5.47 | 37.71 | 87.06 | -0.434 |
| *VvANS21* | *VIT_203s0091g01080.1* | 371 | 41314.38 | 5.74 | 40.92 | 84.07 | -0.265 |
| *VvANS22* | *VIT_203s0017g00620.1* | 352 | 39122.78 | 6.11 | 40.48 | 89.12 | -0.245 |
| *VvANS23* | *VIT_203s0017g00830.1* | 542 | 60542.52 | 6.03 | 37.47 | 92.56 | -0.201 |
| *VvANS24* | *VIT_203s0017g02350.1* | 355 | 39534.78 | 5.38 | 51.29 | 79.07 | -0.298 |
| *VvANS25* | *VIT_204s0008g04920.2* | 362 | 41223.35 | 5.43 | 40.24 | 83.45 | -0.262 |
| *VvANS26* | *VIT_204s0008g04940.1* | 355 | 39675.45 | 6.46 | 45.63 | 89.55 | -0.175 |
| *VvANS27* | *VIT_204s0023g03370.1* | 363 | 40813.38 | 5.37 | 44.25 | 84.33 | -0.444 |
| *VvANS28* | *VIT_204s0044g01520.1* | 375 | 42784.69 | 5.20 | 31.45 | 76.43 | -0.345 |
| *VvANS29* | *VIT_204s0044g01650.1* | 377 | 42812.04 | 6.28 | 33.13 | 77.59 | -0.410 |
| *VvANS30* | *VIT_204s0044g02010.1* | 298 | 32793.57 | 5.23 | 35.22 | 91.61 | -0.115 |
| *VvANS31* | *VIT_205s0077g00520.1* | 339 | 37341.41 | 5.40 | 46.47 | 81.36 | -0.213 |
| *VvANS32* | *VIT_205s0020g01310.1* | 365 | 41464.43 | 5.56 | 42.71 | 85.23 | -0.320 |
| *VvANS33* | *VIT_205s0049g00220.1* | 364 | 41084.89 | 5.36 | 37.64 | 85.38 | -0.275 |
| *VvANS34* | *VIT_205s0049g00230.1* | 307 | 34831.78 | 5.19 | 51.82 | 87.62 | -0.343 |
| *VvANS35* | *VIT_205s0049g00270.1* | 365 | 41438.21 | 5.79 | 41.64 | 79.01 | -0.384 |
| *VvANS36* | *VIT_205s0049g00300.1* | 367 | 41480.44 | 4.98 | 40.49 | 86.05 | -0.251 |
| *VvANS37* | *VIT_205s0049g00310.1* | 363 | 41384.43 | 6.03 | 37.10 | 83.72 | -0.386 |
| *VvANS38* | *VIT_205s0049g00330.1* | 359 | 40279.26 | 6.84 | 41.22 | 88.52 | -0.296 |
| *VvANS39* | *VIT_205s0049g00340.1* | 368 | 41217.32 | 6.90 | 39.58 | 86.09 | -0.307 |
| *VvANS40* | *VIT_205s0049g00350.1* | 368 | 41601.60 | 5.57 | 40.72 | 93.78 | -0.237 |
| *VvANS41* | *VIT_205s0049g00360.1* | 208 | 23213.56 | 5.55 | 41.56 | 92.74 | -0.280 |
| *VvANS42* | *VIT_205s0049g00370.1* | 339 | 38364.89 | 5.47 | 35.76 | 95.43 | -0.255 |
| *VvANS43* | *VIT_205s0049g00390.1* | 373 | 42352.16 | 5.38 | 37.08 | 81.80 | -0.404 |
| *VvANS44* | *VIT_205s0049g00420.1* | 372 | 42106.81 | 5.29 | 44.64 | 89.89 | -0.333 |
| *VvANS45* | *VIT_205s0049g00430.1* | 375 | 42226.03 | 5.25 | 37.02 | 81.84 | -0.368 |
| *VvANS46* | *VIT_206s0004g00760.1* | 358 | 40146.10 | 5.79 | 35.41 | 92.18 | -0.230 |
| *VvANS47* | *VIT_206s0004g06790.1* | 337 | 37888.99 | 5.55 | 37.92 | 86.38 | -0.354 |
| *VvANS48* | *VIT_207s0005g01920.1* | 333 | 37069.67 | 6.34 | 51.38 | 89.82 | -0.174 |
| *VvANS49* | *VIT_207s0005g03060.1* | 377 | 42597.70 | 6.36 | 38.85 | 81.70 | -0.341 |
| *VvANS50* | *VIT_207s0005g03130.1* | 212 | 24057.34 | 6.23 | 31.48 | 80.00 | -0.297 |
| *VvANS51* | *VIT_207s0005g03150.1* | 341 | 38264.67 | 5.55 | 41.05 | 92.02 | -0.250 |
| *VvANS52* | *VIT_208s0007g00750.1* | 701 | 80177.52 | 5.25 | 45.95 | 87.56 | -0.381 |
| *VvANS53* | *VIT_208s0007g03040.1* | 363 | 40918.62 | 6.07 | 36.96 | 85.87 | -0.343 |
| *VvANS54* | *VIT_208s0007g03050.1* | 363 | 40773.47 | 6.02 | 38.30 | 83.44 | -0.340 |
| *VvANS55* | *VIT_208s0007g04540.1* | 377 | 42487.59 | 5.62 | 49.46 | 82.44 | -0.314 |
| *VvANS56* | *VIT_209s0002g05270.1* | 365 | 40156.86 | 6.15 | 41.60 | 91.10 | -0.132 |
| *VvANS57* | *VIT_209s0002g05280.1* | 378 | 43553.73 | 6.76 | 36.65 | 77.91 | -0.476 |
| *VvANS58* | *VIT_209s0002g05290.1* | 345 | 39362.82 | 6.60 | 41.76 | 76.87 | -0.409 |
| *VvANS59* | *VIT_209s0002g05300.1* | 359 | 39184.92 | 5.14 | 52.64 | 94.57 | -0.041 |
| *VvANS60* | *VIT_209s0002g05320.1* | 313 | 35634.69 | 6.09 | 36.06 | 78.50 | -0.310 |
| *VvANS61* | *VIT_209s0002g05340.1* | 316 | 35611.98 | 6.92 | 40.59 | 84.81 | -0.285 |
| *VvANS62* | *VIT_209s0002g05350.2* | 317 | 35749.71 | 6.10 | 36.59 | 80.88 | -0.328 |
| *VvANS63* | *VIT_209s0002g08090.2* | 352 | 39889.59 | 5.17 | 41.01 | 86.39 | -0.374 |
| *VvANS64* | *VIT_210s0116g00410.1* | 421 | 48396.48 | 6.21 | 49.21 | 80.78 | -0.245 |
| *VvANS65* | *VIT_210s0003g02260.1* | 290 | 32756.78 | 6.01 | 37.56 | 87.03 | -0.261 |
| *VvANS66* | *VIT_210s0003g02280.1* | 303 | 34566.20 | 7.03 | 32.50 | 92.94 | -0.232 |
| *VvANS67* | *VIT_210s0003g02290.1* | 359 | 40392.92 | 5.63 | 44.39 | 88.02 | -0.230 |
| *VvANS68* | *VIT_210s0003g02300.1* | 362 | 40943.56 | 5.60 | 45.04 | 91.05 | -0.228 |
| *VvANS69* | *VIT_210s0003g02320.1* | 446 | 51063.54 | 6.05 | 46.21 | 92.69 | -0.068 |
| *VvANS70* | *VIT_210s0003g02330.1* | 359 | 40451.00 | 5.63 | 44.58 | 88.27 | -0.230 |
| *VvANS71* | *VIT_210s0003g02340.1* | 307 | 35042.56 | 5.89 | 41.23 | 85.08 | -0.323 |
| *VvANS72* | *VIT_210s0003g02390.1* | 362 | 40868.53 | 5.68 | 44.80 | 92.65 | -0.175 |
| *VvANS73* | *VIT_210s0003g02410.1* | 365 | 41558.68 | 5.47 | 47.60 | 87.04 | -0.308 |
| *VvANS74* | *VIT_210s0003g02410.2* | 369 | 42006.22 | 5.55 | 51.90 | 84.01 | -0.287 |
| *VvANS75* | *VIT_210s0003g02450.1* | 366 | 41328.61 | 5.38 | 47.22 | 90.00 | -0.200 |
| *VvANS76* | *VIT_210s0003g02470.1* | 214 | 23816.92 | 7.02 | 44.50 | 104.77 | 0.063 |
| *VvANS77* | *VIT_210s0003g02490.1* | 214 | 23720.39 | 5.95 | 34.97 | 91.07 | -0.073 |
| *VvANS78* | *VIT_210s0003g03490.1* | 333 | 37268.60 | 5.42 | 45.89 | 83.33 | -0.288 |
| *VvANS79* | *VIT_211s0016g02380.2* | 329 | 37727.99 | 6.03 | 38.63 | 79.45 | -0.464 |
| *VvANS80* | *VIT_211s0118g00360.5* | 328 | 38015.16 | 7.69 | 28.80 | 98.90 | -0.186 |
| *VvANS81* | *VIT_211s0118g00370.1* | 322 | 36273.33 | 5.06 | 47.26 | 84.10 | -0.313 |
| *VvANS82* | *VIT_211s0118g00390.1* | 356 | 40301.15 | 5.51 | 42.02 | 84.78 | -0.338 |
| *VvANS83* | *VIT_211s0118g00390.6* | 211 | 24260.86 | 6.18 | 28.33 | 92.27 | -0.263 |
| *VvANS84* | *VIT_212s0028g02410.1* | 248 | 28096.29 | 7.00 | 48.24 | 84.48 | -0.286 |
| *VvANS85* | *VIT_212s0028g02420.4* | 384 | 42957.27 | 5.76 | 37.61 | 93.13 | -0.108 |
| *VvANS86* | *VIT_212s0028g02420.1* | 353 | 39570.24 | 6.42 | 32.69 | 85.01 | -0.258 |
| *VvANS87* | *VIT_212s0059g01380.3* | 273 | 30746.43 | 5.93 | 23.04 | 85.71 | -0.347 |
| *VvANS88* | *VIT_212s0059g01380.2* | 234 | 26127.26 | 6.59 | 20.48 | 90.00 | -0.189 |
| *VvANS89* | *VIT_212s0059g01380.1* | 310 | 35013.21 | 5.39 | 21.75 | 82.10 | -0.372 |
| *VvANS90* | *VIT_213s0019g02010.1* | 367 | 41664.67 | 5.39 | 51.49 | 86.54 | -0.408 |
| *VvANS91* | *VIT_213s0047g00210.1* | 337 | 38337.42 | 5.58 | 48.23 | 80.45 | -0.443 |
| *VvANS92* | *VIT_215s0021g00950.1* | 364 | 41345.86 | 5.66 | 44.10 | 95.00 | -0.151 |
| *VvANS93* | *VIT_215s0048g01320.1* | 384 | 43585.77 | 6.60 | 38.76 | 72.34 | -0.339 |
| *VvANS94* | *VIT_215s0048g02430.1* | 344 | 38819.38 | 6.06 | 42.73 | 90.41 | -0.275 |
| *VvANS95* | *VIT_215s0046g02550.1* | 328 | 37048.38 | 5.41 | 50.06 | 80.46 | -0.217 |
| *VvANS96* | *VIT_216s0013g00730.1* | 200 | 22731.33 | 8.36 | 36.22 | 93.10 | -0.257 |
| *VvANS97* | *VIT_216s0013g00740.1* | 355 | 39394.87 | 5.63 | 41.87 | 86.00 | -0.277 |
| *VvANS98* | *VIT_216s0022g00420.1* | 367 | 41800.41 | 5.61 | 53.11 | 91.91 | -0.298 |
| *VvANS99* | *VIT_216s0022g00430.1* | 385 | 43673.43 | 5.51 | 47.87 | 89.40 | -0.277 |
| *VvANS100* | *VIT_216s0022g02310.1* | 382 | 43159.12 | 5.79 | 36.66 | 74.27 | -0.313 |
| *VvANS101* | *VIT_216s0050g00640.1* | 327 | 37015.20 | 5.28 | 55.27 | 75.63 | -0.203 |
| *VvANS102* | *VIT_216s0098g00860.1* | 338 | 38725.30 | 6.14 | 42.20 | 82.75 | -0.428 |
| *VvANS103* | *VIT_218s0001g01390.1* | 378 | 43290.34 | 6.99 | 27.11 | 75.03 | -0.419 |
| *VvANS104* | *VIT_218s0001g01840.1* | 311 | 35219.27 | 5.37 | 30.93 | 82.73 | -0.472 |
| *VvANS105* | *VIT_218s0001g03430.1* | 332 | 37658.07 | 5.32 | 41.24 | 85.06 | -0.422 |
| *VvANS106* | *VIT_218s0001g03470.1* | 335 | 37954.50 | 5.82 | 37.84 | 89.01 | -0.374 |
| *VvANS107* | *VIT_218s0001g03490.1* | 335 | 38392.04 | 6.22 | 40.28 | 84.09 | -0.465 |
| *VvANS108* | *VIT_218s0001g03510.1* | 680 | 77644.16 | 5.73 | 39.62 | 90.13 | -0.355 |
| *VvANS109* | *VIT_218s0001g06690.2* | 344 | 38880.31 | 5.67 | 38.63 | 80.41 | -0.440 |
| *VvANS110* | *VIT_218s0001g06690.3* | 321 | 37053.65 | 9.32 | 46.92 | 91.65 | -0.232 |
| *VvANS111* | *VIT_218s0001g14310.1* | 358 | 40415.03 | 5.40 | 49.29 | 83.04 | -0.449 |
| *VvANS112* | *VIT_219s0177g00030.1* | 329 | 37805.99 | 5.85 | 47.92 | 82.64 | -0.304 |
| *VvANS113* | *VIT_219s0140g00120.1* | 323 | 36116.44 | 6.54 | 51.22 | 83.90 | -0.193 |
| *VvANS114* | *VIT_219s0140g00140.1* | 332 | 37167.71 | 8.22 | 36.23 | 88.34 | -0.211 |
| *VvANS115* | *VIT_219s0027g01110.1* | 366 | 40606.33 | 5.26 | 30.23 | 83.52 | -0.283 |
| *VvANS116* | *VIT_200s0250g00090.1* | 374 | 42247.84 | 6.16 | 56.86 | 80.24 | -0.436 |
| *VVANS117* | *VIT_200s0250g00090.4* | 234 | 26714.62 | 6.96 | 39.90 | 97.82 | -0.215 |
| *VvANS118* | *VIT_200s0521g00010.1* | 366 | 41037.88 | 5.34 | 31.26 | 83.80 | -0.290 |
| *VvANS119* | *VIT_200s0687g00010.1* | 366 | 41034.86 | 5.42 | 28.68 | 83.80 | -0.305 |
| *VvANS120* | *VIT_200s1313g00010.1* | 402 | 45399.13 | 6.20 | 41.26 | 84.63 | -0.265 |
| *VvANS121* | *VIT_200s2086g00010.1* | 205 | 22824.47 | 6.11 | 31.97 | 90.39 | -0.191 |
