# Supplementary material for: Genome-wide identification of grape ANS gene family and expression analysis at different fruit coloration stages
Source: BMC Plant Biol. 2023 Dec 9;23:632. doi: 10.1186/s12870-023-04648-3 (PMC10709965; doi:10.1186/s12870-023-04648-3)
Supplement: Supplementary file 2 — Additional file 2: Supplementary Table S2. VvANS gene codon preference parameter. [file 12870_2023_4648_MOESM2_ESM.docx]

**Supplementary** **Table S2.** *VvANS* gene codon preference parameter

| name | T3s | C3s | A3s | G3s | CAI | CBI | Fop | Nc | GC | GC1 | GC2 | GC3 | GC3s |
| --- | --- | --- | --- | --- | --- | --- | --- | --- | --- | --- | --- | --- | --- |
| *VvANS1* | 0.2993 | 0.4112 | 0.3162 | 0.2412 | 0.189 | -0.012 | 0.407 | 52.05 | 46.93% | 50.13% | 38.13% | 52.53% | 51.40% |
| *VvANS2* | 0.3299 | 0.3866 | 0.3237 | 0.2278 | 0.185 | -0.021 | 0.394 | 55.30 | 43.40% | 47.66% | 34.04% | 48.51% | 48.10% |
| *VvANS3* | 0.3227 | 0.2660 | 0.3358 | 0.3622 | 0.193 | -0.124 | 0.343 | 49.11 | 46.68% | 55.04% | 35.15% | 49.86% | 47.70% |
| *VvANS4* | 0.3251 | 0.2615 | 0.3394 | 0.3622 | 0.193 | -0.141 | 0.333 | 48.96 | 46.50% | 55.04% | 35.15% | 49.32% | 47.30% |
| *VvANS5* | 0.4424 | 0.2274 | 0.3910 | 0.2218 | 0.223 | -0.076 | 0.372 | 53.93 | 41.52% | 51.61% | 36.23% | 36.72% | 34.10% |
| *VvANS6* | 0.2628 | 0.3846 | 0.2500 | 0.3904 | 0.254 | 0.024 | 0.426 | 55.76 | 50.49% | 55.83% | 34.95% | 60.68% | 59.40% |
| *VvANS7* | 0.2212 | 0.4081 | 0.2102 | 0.4139 | 0.218 | 0.058 | 0.451 | 53.79 | 53.05% | 51.52% | 41.62% | 65.99% | 64.70% |
| *VvANS8* | 0.3731 | 0.2692 | 0.3426 | 0.3096 | 0.201 | -0.067 | 0.382 | 49.64 | 46.73% | 53.96% | 39.88% | 46.33% | 44.00% |
| *VvANS9* | 0.3669 | 0.2410 | 0.3511 | 0.3265 | 0.158 | -0.198 | 0.293 | 49.91 | 44.88% | 52.51% | 36.59% | 45.53% | 43.10% |
| *VvANS10* | 0.3380 | 0.3554 | 0.2742 | 0.3017 | 0.211 | 0.008 | 0.412 | 57.22 | 47.37% | 51.58% | 38.11% | 52.44% | 51.00% |
| *VvANS11* | 0.3367 | 0.3605 | 0.2632 | 0.2944 | 0.218 | 0.028 | 0.423 | 55.79 | 48.23% | 52.72% | 39.26% | 52.72% | 51.50% |
| *VvANS12* | 0.4228 | 0.2073 | 0.3739 | 0.2870 | 0.189 | -0.091 | 0.364 | 53.55 | 42.13% | 50.00% | 36.65% | 39.75% | 37.30% |
| *VvANS13* | 0.2885 | 0.4346 | 0.1851 | 0.3969 | 0.227 | 0.015 | 0.421 | 53.29 | 51.31% | 54.49% | 35.39% | 64.04% | 62.90% |
| *VvANS14* | 0.4170 | 0.2713 | 0.3760 | 0.2411 | 0.185 | -0.134 | 0.337 | 54.97 | 41.44% | 51.34% | 30.86% | 42.14% | 38.40% |
| *VvANS15* | 0.4170 | 0.2713 | 0.3760 | 0.2411 | 0.185 | -0.134 | 0.337 | 54.97 | 41.44% | 51.34% | 30.86% | 42.14% | 38.40% |
| *VvANS16* | 0.4100 | 0.2452 | 0.3726 | 0.2562 | 0.196 | -0.104 | 0.350 | 51.72 | 42.94% | 53.60% | 34.58% | 40.63% | 38.10% |
| *VvANS17* | 0.4031 | 0.2752 | 0.3521 | 0.2769 | 0.186 | -0.092 | 0.363 | 54.86 | 43.22% | 52.26% | 33.62% | 43.79% | 41.10% |
| *VvANS18* | 0.3496 | 0.3383 | 0.2421 | 0.3793 | 0.204 | -0.041 | 0.392 | 55.22 | 48.01% | 53.64% | 35.57% | 54.81% | 53.60% |
| *VvANS19* | 0.3397 | 0.3110 | 0.2814 | 0.3757 | 0.186 | -0.057 | 0.380 | 58.70 | 47.30% | 54.04% | 34.93% | 52.94% | 51.70% |
| *VvANS20* | 0.3419 | 0.3259 | 0.2660 | 0.3394 | 0.182 | -0.061 | 0.377 | 55.61 | 48.05% | 52.79% | 38.83% | 52.54% | 51.30% |
| *VvANS21* | 0.3481 | 0.3370 | 0.2500 | 0.3605 | 0.190 | -0.066 | 0.377 | 57.43 | 48.88% | 55.39% | 37.32% | 53.94% | 52.70% |
| *VvANS22* | 0.3520 | 0.3240 | 0.2834 | 0.3304 | 0.194 | -0.025 | 0.395 | 58.17 | 46.85% | 52.44% | 36.28% | 51.83% | 49.70% |
| *VvANS23* | 0.3755 | 0.3234 | 0.2372 | 0.3574 | 0.205 | -0.010 | 0.407 | 54.47 | 47.81% | 54.23% | 36.44% | 52.77% | 51.50% |
| *VvANS24* | 0.3621 | 0.3211 | 0.2703 | 0.3116 | 0.201 | -0.011 | 0.403 | 52.84 | 47.77% | 53.81% | 38.41% | 51.10% | 49.10% |
| *VvANS25* | 0.3510 | 0.3592 | 0.2533 | 0.3349 | 0.215 | -0.017 | 0.403 | 54.28 | 48.67% | 55.91% | 36.10% | 53.99% | 52.50% |
| *VvANS26* | 0.3511 | 0.3053 | 0.2780 | 0.3915 | 0.206 | -0.074 | 0.372 | 48.24 | 46.84% | 54.89% | 33.05% | 52.59% | 51.20% |
| *VvANS27* | 0.3614 | 0.2772 | 0.2776 | 0.3496 | 0.195 | -0.106 | 0.348 | 53.91 | 50.45% | 60.75% | 39.52% | 51.08% | 48.70% |
| *VvANS28* | 0.3262 | 0.3548 | 0.3011 | 0.2749 | 0.194 | -0.055 | 0.376 | 53.68 | 46.93% | 51.84% | 37.96% | 50.99% | 49.40% |
| *VvANS29* | 0.3119 | 0.3257 | 0.3292 | 0.2907 | 0.179 | -0.022 | 0.393 | 55.17 | 46.16% | 50.46% | 37.94% | 50.09% | 48.20% |
| *VvANS30* | 0.3918 | 0.3127 | 0.3015 | 0.2236 | 0.215 | 0.035 | 0.434 | 60.30 | 48.60% | 57.02% | 42.98% | 45.79% | 43.10% |
| *VvANS31* | 0.4197 | 0.2852 | 0.2989 | 0.2263 | 0.211 | 0.026 | 0.417 | 58.19 | 45.00% | 53.17% | 38.57% | 43.25% | 40.80% |
| *VvANS32* | 0.3797 | 0.3458 | 0.2538 | 0.2470 | 0.202 | -0.012 | 0.401 | 56.67 | 49.53% | 57.87% | 41.29% | 49.44% | 47.70% |
| *VvANS33* | 0.2662 | 0.4101 | 0.1864 | 0.4183 | 0.225 | 0.031 | 0.434 | 53.08 | 47.04% | 54.14% | 37.28% | 49.70% | 64.00% |
| *VvANS34* | 0.4276 | 0.2897 | 0.3498 | 0.2450 | 0.233 | -0.036 | 0.404 | 53.93 | 42.91% | 51.33% | 35.11% | 42.29% | 40.20% |
| *VvANS35* | 0.3750 | 0.3125 | 0.2930 | 0.3127 | 0.229 | 0.007 | 0.423 | 55.69 | 46.03% | 51.06% | 37.04% | 50.00% | 47.60% |
| *VvANS36* | 0.2769 | 0.3926 | 0.2478 | 0.3178 | 0.226 | 0.067 | 0.448 | 57.63 | 52.73% | 58.86% | 40.47% | 58.86% | 57.00% |
| *VvANS37* | 0.3381 | 0.3203 | 0.2778 | 0.3190 | 0.204 | -0.039 | 0.392 | 51.94 | 48.53% | 52.65% | 41.76% | 51.18% | 49.80% |
| *VvANS38* | 0.3509 | 0.2772 | 0.3296 | 0.3279 | 0.177 | -0.126 | 0.341 | 53.94 | 45.72% | 51.91% | 37.16% | 48.09% | 45.80% |
| *VvANS39* | 0.3090 | 0.3160 | 0.3296 | 0.3203 | 0.197 | -0.033 | 0.391 | 60.52 | 48.04% | 56.71% | 36.16% | 51.23% | 49.40% |
| *VvANS40* | 0.3782 | 0.2605 | 0.3289 | 0.3160 | 0.179 | -0.137 | 0.330 | 59.64 | 46.21% | 55.84% | 36.69% | 46.10% | 43.90% |
| *VvANS41* | 0.3581 | 0.2770 | 0.3580 | 0.2905 | 0.196 | -0.048 | 0.386 | 58.40 | 45.54% | 53.55% | 37.16% | 45.90% | 43.40% |
| *VvANS42* | 0.3699 | 0.3116 | 0.3030 | 0.2960 | 0.211 | -0.011 | 0.405 | 59.39 | 46.92% | 56.79% | 35.05% | 48.91% | 46.70% |
| *VvANS43* | 0.3426 | 0.2803 | 0.3678 | 0.2915 | 0.184 | -0.099 | 0.351 | 58.88 | 45.42% | 54.12% | 35.99% | 46.15% | 44.00% |
| *VvANS44* | 0.4316 | 0.2281 | 0.3745 | 0.2398 | 0.230 | -0.057 | 0.380 | 52.46 | 42.50% | 53.06% | 36.39% | 38.06% | 35.70% |
| *VvANS45* | 0.4320 | 0.2279 | 0.3838 | 0.2289 | 0.232 | -0.051 | 0.386 | 52.20 | 42.55% | 52.85% | 37.40% | 37.40% | 34.90% |
| *VvANS46* | 0.3767 | 0.2534 | 0.3832 | 0.2767 | 0.185 | -0.112 | 0.345 | 55.91 | 43.45% | 53.93% | 34.96% | 41.46% | 40.10% |
| *VvANS47* | 0.3392 | 0.2515 | 0.4423 | 0.2245 | 0.184 | -0.067 | 0.360 | 53.42 | 44.50% | 55.98% | 38.76% | 38.76% | 37.40% |
| *VvANS48* | 0.4511 | 0.2218 | 0.3557 | 0.2756 | 0.210 | -0.068 | 0.372 | 56.02 | 41.18% | 53.24% | 32.35% | 37.94% | 36.60% |
| *VvANS49* | 0.3904 | 0.2877 | 0.3657 | 0.2430 | 0.206 | -0.012 | 0.409 | 55.67 | 44.03% | 53.21% | 35.83% | 43.05% | 40.60% |
| *VvANS50* | 0.3733 | 0.2600 | 0.4059 | 0.2490 | 0.187 | -0.083 | 0.366 | 55.28 | 42.45% | 50.94% | 36.19% | 40.21% | 38.50% |
| *VvANS51* | 0.3493 | 0.3082 | 0.3553 | 0.2706 | 0.187 | -0.086 | 0.369 | 54.04 | 46.19% | 55.85% | 35.90% | 46.81% | 44.40% |
| *VvANS52* | 0.3679 | 0.2571 | 0.2704 | 0.3840 | 0.193 | -0.145 | 0.323 | 48.62 | 46.98% | 54.87% | 35.38% | 50.70% | 48.80% |
| *VvANS53* | 0.3660 | 0.2830 | 0.2791 | 0.3361 | 0.188 | -0.083 | 0.363 | 57.51 | 47.04% | 54.14% | 37.28% | 49.70% | 48.00% |
| *VvANS54* | 0.3091 | 0.3491 | 0.2550 | 0.3261 | 0.159 | -0.103 | 0.344 | 51.83 | 50.00% | 52.99% | 41.92% | 55.09% | 53.40% |
| *VvANS55* | 0.3682 | 0.2365 | 0.3094 | 0.3828 | 0.202 | -0.043 | 0.394 | 54.10 | 46.38% | 52.91% | 38.10% | 48.15% | 46.30% |
| *VvANS56* | 0.3793 | 0.3621 | 0.2733 | 0.2555 | 0.229 | 0.049 | 0.449 | 53.82 | 46.17% | 50.70% | 38.50% | 49.30% | 47.80% |
| *VvANS57* | 0.3993 | 0.2698 | 0.3242 | 0.2766 | 0.194 | 0.011 | 0.413 | 53.03 | 44.74% | 53.51% | 37.72% | 42.98% | 41.90% |
| *VvANS58* | 0.3703 | 0.2462 | 0.3809 | 0.3077 | 0.192 | -0.121 | 0.345 | 53.20 | 43.11% | 51.57% | 33.76% | 44.02% | 41.20% |
| *VvANS59* | 0.2882 | 0.3472 | 0.2372 | 0.4096 | 0.226 | 0.021 | 0.426 | 57.49 | 50.18% | 53.02% | 38.46% | 59.07% | 57.70% |
| *VvANS60* | 0.2847 | 0.3701 | 0.2288 | 0.4073 | 0.240 | 0.055 | 0.450 | 54.62 | 49.54% | 50.55% | 37.36% | 60.71% | 59.10% |
| *VvANS61* | 0.3946 | 0.3043 | 0.3000 | 0.2908 | 0.187 | -0.059 | 0.380 | 56.47 | 44.18% | 48.94% | 36.51% | 47.09% | 45.20% |
| *VvANS62* | 0.2736 | 0.4495 | 0.2370 | 0.2500 | 0.226 | 0.087 | 0.457 | 53.64 | 53.55% | 58.74% | 42.35% | 59.56% | 57.70% |
| *VvANS63* | 0.4091 | 0.2727 | 0.3796 | 0.2549 | 0.225 | -0.015 | 0.415 | 51.86 | 42.13% | 50.92% | 34.04% | 41.42% | 39.30% |
| *VvANS64* | 0.3704 | 0.2926 | 0.4234 | 0.2096 | 0.193 | -0.094 | 0.373 | 52.21 | 42.29% | 48.27% | 38.15% | 40.46% | 38.30% |
| *VvANS65* | 0.3333 | 0.4167 | 0.2509 | 0.2205 | 0.249 | 0.086 | 0.456 | 57.27 | 51.39% | 58.61% | 41.94% | 53.61% | 51.70% |
| *VvANS66* | 0.4413 | 0.2470 | 0.3935 | 0.2256 | 0.199 | -0.127 | 0.351 | 55.41 | 41.08% | 50.32% | 35.03% | 37.90% | 35.10% |
| *VvANS67* | 0.3911 | 0.2742 | 0.3783 | 0.2300 | 0.171 | -0.146 | 0.329 | 52.47 | 43.53% | 52.05% | 36.91% | 41.64% | 38.90% |
| *VvANS68* | 0.4938 | 0.2469 | 0.3043 | 0.2500 | 0.215 | -0.068 | 0.385 | 52.83 | 42.24% | 51.57% | 34.91% | 40.25% | 37.50% |
| *VvANS69* | 0.4023 | 0.2218 | 0.4170 | 0.2410 | 0.195 | -0.084 | 0.366 | 52.85 | 42.12% | 52.41% | 36.54% | 37.39% | 35.10% |
| *VvANS70* | 0.3242 | 0.3639 | 0.3032 | 0.2941 | 0.233 | 0.071 | 0.458 | 54.39 | 46.52% | 49.05% | 38.15% | 52.37% | 50.50% |
| *VvANS71* | 0.3304 | 0.2775 | 0.3571 | 0.3144 | 0.174 | -0.090 | 0.358 | 52.75 | 44.56% | 50.52% | 35.05% | 48.11% | 45.30% |
| *VvANS72* | 0.3277 | 0.2647 | 0.3641 | 0.3283 | 0.159 | -0.078 | 0.358 | 53.64 | 44.30% | 51.97% | 32.89% | 48.03% | 44.90% |
| *VvANS73* | 0.3396 | 0.2687 | 0.3455 | 0.3214 | 0.203 | -0.052 | 0.378 | 56.46 | 45.65% | 52.78% | 36.11% | 48.06% | 45.10% |
| *VvANS74* | 0.3493 | 0.2757 | 0.3491 | 0.3068 | 0.197 | -0.046 | 0.379 | 55.07 | 45.00% | 52.89% | 34.99% | 47.11% | 44.30% |
| *VvANS75* | 0.3343 | 0.2878 | 0.3634 | 0.2951 | 0.197 | -0.020 | 0.393 | 54.29 | 44.22% | 50.34% | 35.35% | 46.98% | 44.50% |
| *VvANS76* | 0.3371 | 0.2772 | 0.3370 | 0.3241 | 0.199 | -0.051 | 0.378 | 54.11 | 45.65% | 52.22% | 35.83% | 48.89% | 46.00% |
| *VvANS77* | 0.3540 | 0.2522 | 0.3656 | 0.3286 | 0.201 | -0.031 | 0.394 | 55.48 | 43.83% | 51.30% | 33.44% | 46.75% | 43.60% |
| *VvANS78* | 0.3480 | 0.2674 | 0.3574 | 0.3056 | 0.200 | -0.033 | 0.387 | 58.14 | 44.90% | 53.17% | 35.26% | 46.28% | 43.60% |
| *VvANS79* | 0.4022 | 0.2681 | 0.3173 | 0.3040 | 0.188 | -0.105 | 0.352 | 52.84 | 44.44% | 53.28% | 34.15% | 45.90% | 43.20% |
| *VvANS80* | 0.3732 | 0.3043 | 0.3162 | 0.3016 | 0.188 | -0.104 | 0.355 | 56.47 | 45.68% | 53.78% | 34.59% | 48.65% | 45.80% |
| *VvANS81* | 0.3768 | 0.2782 | 0.3236 | 0.2840 | 0.191 | -0.047 | 0.379 | 55.74 | 45.69% | 54.50% | 36.24% | 46.32% | 43.70% |
| *VvANS82* | 0.3699 | 0.3006 | 0.3253 | 0.2288 | 0.174 | -0.063 | 0.356 | 56.76 | 45.89% | 56.28% | 36.74% | 44.65% | 42.40% |
| *VvANS83* | 0.4201 | 0.2308 | 0.3072 | 0.3165 | 0.171 | -0.108 | 0.353 | 56.78 | 42.79% | 48.84% | 34.88% | 44.65% | 41.30% |
| *VvANS84* | 0.3118 | 0.3346 | 0.2560 | 0.3766 | 0.219 | 0.047 | 0.439 | 50.10 | 48.60% | 51.80% | 38.02% | 55.99% | 54.50% |
| *VvANS85* | 0.3920 | 0.2840 | 0.3390 | 0.3009 | 0.201 | -0.147 | 0.341 | 55.81 | 43.64% | 50.30% | 34.55% | 46.06% | 43.30% |
| *VvANS86* | 0.3992 | 0.2171 | 0.3967 | 0.2851 | 0.177 | -0.131 | 0.327 | 49.47 | 39.82% | 48.94% | 31.31% | 39.21% | 37.40% |
| *VvANS87* | 0.3953 | 0.2171 | 0.3629 | 0.2814 | 0.177 | -0.126 | 0.329 | 51.53 | 44.79% | 55.73% | 38.08% | 40.56% | 38.70% |
| *VvANS88* | 0.3897 | 0.2059 | 0.4066 | 0.2706 | 0.173 | -0.127 | 0.333 | 53.38 | 43.04% | 54.06% | 36.13% | 38.94% | 36.50% |
| *VvANS89* | 0.3688 | 0.2313 | 0.3976 | 0.2611 | 0.179 | -0.156 | 0.320 | 53.70 | 44.97% | 55.66% | 38.21% | 41.04% | 38.40% |
| *VvANS90* | 0.3906 | 0.2135 | 0.3969 | 0.2554 | 0.183 | -0.127 | 0.333 | 49.96 | 44.31% | 55.82% | 38.15% | 38.96% | 36.70% |
| *VvANS91* | 0.3929 | 0.2338 | 0.3624 | 0.2704 | 0.204 | -0.039 | 0.386 | 58.77 | 44.07% | 54.29% | 36.62% | 41.30% | 39.20% |
| *VvANS92* | 0.3684 | 0.2491 | 0.3626 | 0.2846 | 0.182 | -0.105 | 0.352 | 57.52 | 45.10% | 53.67% | 38.42% | 43.22% | 41.30% |
| *VvANS93* | 0.2282 | 0.4660 | 0.3015 | 0.3105 | 0.257 | 0.039 | 0.439 | 52.82 | 50.12% | 57.66% | 32.12% | 60.58% | 59.20% |
| *VvANS94* | 0.2541 | 0.4486 | 0.2994 | 0.2893 | 0.255 | 0.037 | 0.434 | 56.36 | 49.36% | 56.60% | 33.19% | 58.30% | 57.10% |
| *VvANS95* | 0.2445 | 0.4585 | 0.2928 | 0.3223 | 0.252 | 0.008 | 0.425 | 52.79 | 49.84% | 56.91% | 31.83% | 60.77% | 58.80% |
| *VvANS96* | 0.3704 | 0.2556 | 0.3653 | 0.3147 | 0.230 | -0.048 | 0.389 | 51.59 | 43.57% | 52.45% | 32.61% | 45.65% | 42.70% |
| *VvANS97* | 0.4082 | 0.2884 | 0.2922 | 0.2996 | 0.191 | -0.125 | 0.345 | 57.12 | 45.86% | 52.66% | 38.17% | 46.75% | 44.60% |
| *VvANS98* | 0.3489 | 0.2914 | 0.3594 | 0.2692 | 0.192 | -0.040 | 0.378 | 54.90 | 45.11% | 55.34% | 34.52% | 45.48% | 43.30% |
| *VvANS99* | 0.3562 | 0.3333 | 0.3109 | 0.2835 | 0.198 | -0.054 | 0.388 | 54.45 | 46.67% | 50.65% | 39.48% | 49.87% | 47.50% |
| *VvANS100* | 0.3310 | 0.3627 | 0.2691 | 0.3114 | 0.203 | -0.008 | 0.403 | 55.38 | 47.92% | 52.46% | 37.97% | 53.33% | 51.90% |
| *VvANS101* | 0.4118 | 0.2431 | 0.3320 | 0.3009 | 0.235 | 0.054 | 0.451 | 46.88 | 43.06% | 48.63% | 37.08% | 43.47% | 41.30% |
| *VvANS102* | 0.3590 | 0.2949 | 0.3605 | 0.2500 | 0.193 | -0.013 | 0.402 | 52.50 | 43.62% | 49.25% | 36.32% | 45.27% | 42.30% |
| *VvANS103* | 0.4036 | 0.2250 | 0.3521 | 0.2857 | 0.198 | -0.085 | 0.368 | 55.25 | 44.29% | 52.25% | 38.76% | 41.85% | 39.50% |
| *VvANS104* | 0.3782 | 0.2655 | 0.3273 | 0.3145 | 0.185 | -0.048 | 0.379 | 54.99 | 43.93% | 50.54% | 34.51% | 46.74% | 43.60% |
| *VvANS105* | 0.4041 | 0.2500 | 0.3229 | 0.3050 | 0.196 | -0.053 | 0.380 | 56.59 | 43.70% | 50.26% | 35.75% | 45.08% | 41.90% |
| *VvANS106* | 0.3179 | 0.3609 | 0.2937 | 0.3164 | 0.237 | 0.066 | 0.460 | 49.27 | 48.04% | 51.70% | 38.38% | 54.05% | 52.10% |
| *VvANS107* | 0.3755 | 0.3083 | 0.3304 | 0.2897 | 0.227 | -0.007 | 0.421 | 53.08 | 43.19% | 45.73% | 36.28% | 47.56% | 45.00% |
| *VvANS108* | 0.3473 | 0.3130 | 0.3102 | 0.3304 | 0.176 | -0.168 | 0.314 | 52.45 | 46.51% | 52.80% | 35.99% | 50.74% | 48.60% |
| *VvANS109* | 0.3709 | 0.3046 | 0.3271 | 0.2840 | 0.224 | -0.004 | 0.415 | 52.34 | 46.26% | 52.51% | 38.79% | 47.49% | 45.30% |
| *VvANS110* | 0.2435 | 0.4087 | 0.2554 | 0.4186 | 0.242 | 0.023 | 0.431 | 49.89 | 50.32% | 55.45% | 32.69% | 62.82% | 61.50% |
| *VvANS111* | 0.3684 | 0.3198 | 0.2550 | 0.3500 | 0.218 | -0.057 | 0.374 | 58.50 | 48.65% | 58.86% | 33.93% | 53.15% | 51.30% |
| *VvANS112* | 0.3755 | 0.3360 | 0.1829 | 0.4085 | 0.242 | -0.021 | 0.399 | 52.54 | 48.91% | 53.87% | 35.12% | 57.74% | 56.00% |
| *VvANS113* | 0.3779 | 0.3282 | 0.2276 | 0.3616 | 0.203 | -0.083 | 0.360 | 56.41 | 48.02% | 53.87% | 36.61% | 53.57% | 51.90% |
| *VvANS114* | 0.3524 | 0.3448 | 0.2300 | 0.3726 | 0.208 | -0.067 | 0.368 | 50.24 | 47.38% | 51.25% | 35.54% | 55.36% | 54.00% |
| *VvANS115* | 0.3396 | 0.2761 | 0.4057 | 0.2763 | 0.168 | -0.114 | 0.346 | 56.24 | 46.16% | 50.46% | 37.94% | 50.09% | 41.90% |
| *VvANS116* | 0.3551 | 0.2776 | 0.3744 | 0.3054 | 0.173 | -0.072 | 0.364 | 56.32 | 41.82% | 46.58% | 32.61% | 46.27% | 43.00% |
| *VvANS117* | 0.2788 | 0.3271 | 0.2310 | 0.4466 | 0.200 | -0.047 | 0.390 | 53.86 | 52.74% | 59.05% | 38.16% | 61.00% | 59.60% |
| *VvANS118* | 0.4055 | 0.2638 | 0.3471 | 0.3077 | 0.218 | -0.029 | 0.407 | 53.83 | 42.73% | 48.79% | 36.36% | 43.03% | 41.90% |
| *VvANS119* | 0.3525 | 0.2989 | 0.3361 | 0.2752 | 0.187 | -0.032 | 0.394 | 49.39 | 44.44% | 47.53% | 39.20% | 46.60% | 44.50% |
| *VvANS120* | 0.3396 | 0.3022 | 0.3240 | 0.2969 | 0.178 | -0.048 | 0.383 | 53.44 | 43.74% | 44.74% | 38.14% | 48.35% | 46.40% |
| *VvANS121* | 0.3050 | 0.2801 | 0.2986 | 0.3923 | 0.203 | -0.080 | 0.369 | 52.28 | 48.50% | 55.59% | 36.24% | 53.68% | 51.70% |
| Average | 0.3580 | 0.3008 | 0.3187 | 0.3041 | 0.202 | -0.050 | 0.384 | 54.30 | 45.91% | 52.95% | 36.50% | 48.30% | 46.29% |
