# Supplementary material for: Genome-wide identification of grape ANS gene family and expression analysis at different fruit coloration stages
Source: BMC Plant Biol. 2023 Dec 9;23:632. doi: 10.1186/s12870-023-04648-3 (PMC10709965; doi:10.1186/s12870-023-04648-3)
Supplement: Supplementary file 3 — Additional file 3: Supplementary Table S3. Analysis of secondary structure and subcellular localization of VvANS protein. [file 12870_2023_4648_MOESM3_ESM.docx]

**Supplementary** **Table S3.** Analysis of secondary structure and subcellular localization of VvANS protein

| gene | Alpha helix | Random coil | Extended strand | subcellular location |
| --- | --- | --- | --- | --- |
| VvANS1 | 31.30% | 48.60% | 20.10% | chloroplasts、mitochondria、cytoplasm、endoplasmic reticulum、peroxisome |
| VvANS2 | 35.59% | 50.59% | 13.82% | nucleus、cytoplasm nucleus、cytoplasm、peroxisome |
| VvANS3 | 27.73% | 50.70% | 21.57% | cytoplasm、nucleus、extracellular、endoplasmic reticulum vacuole |
| VvANS4 | 25.29% | 58.91% | 15.80% | cytoplasm、nucleus、chloroplasts、extracellular、cytoskeleton |
| VvANS5 | 26.72% | 59.20% | 14.08% | nucleus、cytoplasm、extracellular、cytoskeleton |
| VvANS6 | 38.32% | 45.17% | 16.51% | nucleus、cytoplasm、chloroplasts、extracellular、cytoskeleton |
| VvANS7 | 33.52% | 50.99% | 15.49% | chloroplasts、mitochondria、 cytoplasm、nucleus、peroxisome |
| VvANS8 | 43.15% | 41.96% | 14.88% | cytoplasm、nucleus、cytoskeleton、chloroplasts |
| VvANS9 | 39.09% | 43.63% | 17.28% | cytoplasm、chloroplasts、nucleus、cytoskeleton、endoplasmic reticulum vacuole |
| VvANS10 | 35.26% | 48.84% | 15.90% | chloroplasts、cytoskeleton、cytoplasm、plasma membrane、nucleus |
| VvANS11 | 41.36% | 45.89% | 12.75% | nucleus、cytoplasm、cytoskeleton |
| VvANS12 | 29.24% | 49.12% | 21.64% | cytoplasm、endoplasmic reticulum vacuole |
| VvANS13 | 32.10% | 48.34% | 19.56% | cytoplasm、mitochondria、chloroplasts、extracellular、endoplasmic reticulum、cytoskeleton |
| VvANS14 | 26.21% | 52.93% | 20.87% | cytoplasm、chloroplasts、nucleus、peroxisome、endoplasmic reticulum vacuole |
| VvANS15 | 30.99% | 47.66% | 21.35% | cytoplasm、 chloroplasts、endoplasmic reticulum  vacuole |
| VvANS16 | 31.19% | 48.32% | 20.49% | cytoplasm、cytoskeleton、chloroplasts、nucleus |
| VvANS17 | 26.32% | 49.71% | 23.98% | cytoplasm、chloroplasts、endoplasmic reticulum  vacuole |
| VvANS18 | 29.15% | 48.64% | 22.20% | cytoplasm、nucleus、chloroplasts、plasma membrane、peroxisome、 endoplasmic reticulum vacuole |
| VvANS19 | 27.88% | 49.68% | 22.44% | cytoplasm、 endoplasmic reticulum vacuole |
| VvANS20 | 34.58% | 46.40% | 19.02% | cytoskeleton、cytoplasm、extracellular |
| VvANS21 | 37.20% | 48.52% | 14.29% | cytoplasm、nucleus、extracellular |
| VvANS22 | 27.27% | 53.41% | 19.32% | cytoplasm、nucleus、chloroplasts |
| VvANS23 | 29.52% | 49.08% | 21.40% | cytoplasm、nucleus、peroxisome |
| VvANS24 | 18.59% | 56.06% | 25.35% | cytoplasm、nucleus、vacuole、cytoskeleton |
| VvANS25 | 34.25% | 47.51% | 18.23% | cytoplasm、mitochondria、plasma membrane、chloroplasts、nucleus、extracellular |
| VvANS26 | 35.21% | 49.30% | 15.49% | chloroplasts、cytoplasm、nucleus、extracellular、vacuole、endoplasmic reticulum |
| VvANS27 | 32.78% | 48.48% | 18.73% | cytoplasm、chloroplasts、nucleus、cytoskeleton、endoplasmic reticulum |
| VvANS28 | 45.33% | 42.13% | 12.53% | cytoplasm、mitochondria、chloroplasts、plasma membrane、peroxisome |
| VvANS29 | 32.63% | 49.34% | 18.04% | nucleus、cytoplasm、cytoskeleton、chloroplasts |
| VvANS30 | 39.93% | 44.63% | 15.44% | cytoplasm、chloroplasts、nucleus、cytoskeleton、 endoplasmic reticulum vacuole |
| VvANS31 | 26.25% | 56.34% | 17.40% | nucleus、cytoplasm、cytoskeleton、chloroplasts、endoplasmic reticulum vacuole |
| VvANS32 | 33.42% | 48.49% | 18.08% | cytoplasm、cytoskeleton、nucleus、vacuole、endoplasmic reticulum vacuole |
| VvANS33 | 41.48% | 44.78% | 13.74% | cytoplasm、nucleus、extracellular |
| VvANS34 | 39.41% | 48.86% | 11.73% | nucleus、cytoplasm、cytoskeleton |
| VvANS35 | 29.32% | 56.99% | 13.70% | nucleus、cytoskeleton、cytoplasm、plasma membrane、vacuole |
| VvANS36 | 34.88% | 49.05% | 16.08% | cytoplasm、chloroplasts、nucleus、cytoskeleton、plasma membrane |
| VvANS37 | 33.61% | 51.52% | 14.88% | nucleus、cytoplasm nucleus、chloroplasts、cytoplasm、cytoskeleton |
| VvANS38 | 37.60% | 45.96% | 16.43% | cytoskeleton、cytoplasm、nucleus、endoplasmic reticulum vacuole |
| VvANS39 | 34.78% | 48.64% | 16.58% | cytoplasm、cytoskeleton、nucleus、chloroplasts |
| VvANS40 | 39.40% | 48.10% | 12.50% | cytoplasm、nucleus、chloroplasts、extracellular、cytoskeleton |
| VvANS41 | 29.81% | 54.81% | 15.38% | cytoplasm、nucleus、extracellular |
| VvANS42 | 35.69% | 45.72% | 18.58% | cytoplasm、cytoskeleton、nucleus、extracellular、Golgi apparatus |
| VvANS43 | 41.55% | 49.87% | 8.58% | nucleus、nucleus plasma membrane、cytoplasm、chloroplasts、extracellular、vacuole、peroxisome |
| VvANS44 | 33.33% | 50.27% | 16.40% | nucleus、cytoplasm、chloroplasts、vacuole、peroxisome、Golgi apparatus |
| VvANS45 | 34.40% | 47.73% | 17.87% | cytoplasm、cytoskeleton、nucleus、plasma membrane、peroxisome、Golgi apparatus |
| VvANS46 | 29.61% | 49.44% | 20.95% | peroxisome、cytoplasm、nucleus |
| VvANS47 | 21.96% | 54.30% | 23.74% | cytoplasm、chloroplasts、nucleus、extracellular |
| VvANS48 | 30.03% | 52.55% | 17.42% | nucleus、cytoplasm、chloroplasts、plasma membrane、extracellular |
| VvANS49 | 39.26% | 46.95% | 13.79% | cytoplasm、nucleus、cytoskeleton、mitochondria、plasma membrane |
| VvANS50 | 20.28% | 55.19% | 24.53% | cytoplasm、nucleus、plasma membrane、cytoskeleton plasma membrane、chloroplasts、mitochondria |
| VvANS51 | 24.05% | 57.18% | 18.77% | chloroplasts、nucleus、cytoplasm、mitochondria、cytoplasm mitochondria、plasma membrane |
| VvANS52 | 32.81% | 49.50% | 17.69% | nucleus、cytoplasm、chloroplasts、cytoskeleton、plasma membrane |
| VvANS53 | 27.82% | 52.34% | 19.83% | cytoskeleton、nucleus、cytoplasm |
| VvANS54 | 33.06% | 50.41% | 16.53% | nucleus、cytoplasm、chloroplasts、vacuole |
| VvANS55 | 27.85% | 55.97% | 16.18% | nucleus、chloroplasts、cytoplasm |
| VvANS56 | 36.16% | 48.77% | 15.07% | mitochondria、chloroplasts、nucleus、cytoplasm、cytoskeleton |
| VvANS57 | 38.62% | 47.88% | 13.49% | nucleus、cytoplasm、chloroplasts、plasma membrane |
| VvANS58 | 30.14% | 49.57% | 20.29% | chloroplasts、nucleus、mitochondria |
| VvANS59 | 39.28% | 47.91% | 12.81% | cytoplasm、nucleus、nucleus plasma membrane、chloroplasts、mitochondria |
| VvANS60 | 36.42% | 51.76% | 11.82% | cytoplasm、cytoskeleton、nucleus、peroxisome、Golgi apparatus |
| VvANS61 | 36.08% | 52.53% | 11.39% | cytoplasm、chloroplasts、plasma membrane、nucleus、mitochondria、peroxisome |
| VvANS62 | 29.97% | 50.16% | 19.87% | nucleus、cytoplasm、peroxisome、chloroplasts、extracellular、vacuole |
| VvANS63 | 44.32% | 44.60% | 11.08% | nucleus、cytoplasm、cytoskeleton、chloroplasts、plasma membrane、Golgi apparatus |
| VvANS64 | 35.15% | 44.89% | 19.95% | chloroplasts、nucleus、cytoplasm |
| VvANS65 | 37.93% | 49.66% | 12.41% | cytoplasm、nucleus、chloroplasts、plasma membrane、cytoskeleton、Golgi apparatus |
| VvANS66 | 41.91% | 44.55% | 13.53% | cytoplasm、chloroplasts、mitochondria、extracellular、peroxisome |
| VvANS67 | 34.82% | 51.53% | 13.65% | chloroplasts、cytoplasm、mitochondria、plasma membrane |
| VvANS68 | 34.81% | 51.93% | 13.26% | chloroplasts、cytoplasm、mitochondria、nucleus、plasma membrane、extracellular、peroxisome |
| VvANS69 | 34.30% | 49.10% | 16.59% | Golgi apparatus、cytoplasm、nucleus、chloroplasts、plasma membrane、vacuole |
| VvANS70 | 34.54% | 51.81% | 13.65% | chloroplasts、cytoplasm、nucleus、mitochondria、plasma membrane、extracellular |
| VvANS71 | 35.83% | 47.88% | 16.29% | nucleus、cytoplasm、plasma membrane、extracellular、peroxisome、cytoskeleton |
| VvANS72 | 33.15% | 52.49% | 14.36% | cytoplasm、chloroplasts、nucleus、mitochondria、plasma membrane、extracellular、Golgi apparatus |
| VvANS73 | 40.27% | 43.56% | 16.16% | cytoplasm、nucleus、peroxisome、cytoskeleton、Golgi apparatus |
| VvANS74 | 42.28% | 42.28% | 15.45% | cytoplasm、nucleus、mitochondria、peroxisome、cytoskeleton |
| VvANS75 | 43.44% | 45.08% | 11.48% | cytoplasm、chloroplasts、nucleus |
| VvANS76 | 39.72% | 44.39% | 15.89% | mitochondria、chloroplasts、nucleus、cytoplasm、endoplasmic reticulum |
| VvANS77 | 39.72% | 45.79% | 14.49% | nucleus、cytoplasm、chloroplasts、cytoskeleton |
| VvANS78 | 25.53% | 51.05% | 23.42% | nucleus、cytoplasm、chloroplasts |
| VvANS79 | 37.99% | 45.59% | 16.41% | chloroplasts、cytoplasm、nucleus、mitochondria、endoplasmic reticulum |
| VvANS80 | 40.55% | 40.85% | 18.60% | cytoplasm、cytoplasm endoplasmic reticulum、endoplasmic reticulum、chloroplasts、nucleus、mitochondria、plasma membrane、cytoskeleton |
| VvANS81 | 34.78% | 50.00% | 15.22% | chloroplasts、nucleus、cytoplasm、extracellular、mitochondria、vacuole、endoplasmic reticulum |
| VvANS82 | 30.06% | 53.65% | 16.29% | peroxisome、nucleus、cytoplasm |
| VvANS83 | 36.97% | 44.55% | 18.48% | cytoplasm、chloroplasts、nucleus、extracellular、vacuole、cytoskeleton |
| VvANS84 | 37.90% | 47.58% | 14.52% | cytoplasm、chloroplasts |
| VvANS85 | 43.75% | 41.67% | 14.58% | chloroplasts、cytoplasm、plasma membrane、vacuole、nucleus、cytoskeleton nucleus、mitochondria、endoplasmic reticulum |
| VvANS86 | 37.68% | 47.03% | 15.30% | cytoplasm、chloroplasts、nucleus、plasma membrane、peroxisome、cytoskeleton |
| VvANS87 | 40.29% | 43.59% | 16.12% | cytoplasm、mitochondria、chloroplasts、plasma membrane、endoplasmic reticulum |
| VvANS88 | 33.33% | 46.58% | 20.09% | cytoplasm、cytoplasm endoplasmic reticulum、chloroplasts、nucleus |
| VvANS89 | 40.32% | 44.52% | 15.16% | cytoskeleton、cytoplasm、cytoplasm endoplasmic reticulum、chloroplasts、nucleus |
| VvANS90 | 40.33% | 44.41% | 15.26% | cytoplasm、nucleus、cytoskeleton、plasma membrane、endoplasmic reticulum vacuole |
| VvANS91 | 38.58% | 45.99% | 15.43% | cytoplasm、nucleus、cytoskeleton、vacuole |
| VvANS92 | 43.68% | 45.60% | 10.71% | cytoplasm、chloroplasts、nucleus、plasma membrane、cytoskeleton、endoplasmic reticulum vacuole |
| VvANS93 | 34.90% | 50.52% | 14.58% | nucleus、cytoplasm、chloroplasts、mitochondria、plasma membrane、cytoskeleton |
| VvANS94 | 40.99% | 46.51% | 12.50% | cytoplasm、nucleus、plasma membrane |
| VvANS95 | 26.83% | 46.65% | 26.52% | nucleus、cytoplasm、chloroplasts、extracellular、cytoskeleton |
| VvANS96 | 41.00% | 46.00% | 13.00% | cytoplasm、nucleus、extracellular、peroxisome、cytoskeleton |
| VvANS97 | 42.54% | 45.07% | 12.39% | nucleus、cytoplasm、chloroplasts、vacuole |
| VvANS98 | 35.69% | 49.05% | 15.26% | chloroplasts、nucleus、vacuole、cytoplasm、peroxisome、Golgi apparatus、mitochondria plasma membrane |
| VvANS99 | 31.17% | 52.21% | 16.62% | chloroplasts、nucleus、cytoplasm、vacuole、peroxisome、Golgi apparatus、mitochondria plasma membrane |
| VvANS100 | 37.70% | 48.69% | 13.61% | nucleus、plasma membrane、vacuole、cytoskeleton |
| VvANS101 | 27.83% | 48.32% | 23.85% | nucleus、chloroplasts、cytoplasm、extracellular |
| VvANS102 | 39.05% | 46.15% | 14.79% | chloroplasts、peroxisome、cytoplasm、nucleus |
| VvANS103 | 37.30% | 48.68% | 14.02% | nucleus、cytoplasm、cytoskeleton、plasma membrane、endoplasmic reticulum vacuole |
| VvANS104 | 34.41% | 45.34% | 20.26% | cytoplasm、cytoplasm endoplasmic reticulum、cytoskeleton、nucleus、Golgi apparatus |
| VvANS105 | 29.82% | 53.61% | 16.57% | cytoplasm、chloroplasts、extracellular、cytoskeleton、endoplasmic reticulum vacuole |
| VvANS106 | 25.37% | 56.12% | 18.51% | cytoplasm、mitochondria 、extracellular、chloroplasts、nucleus、vacuole、endoplasmic reticulum |
| VvANS107 | 30.15% | 52.54% | 17.31% | cytoplasm、cytoskeleton、nucleus、endoplasmic reticulum vacuole |
| VvANS108 | 34.56% | 49.56% | 15.88% | cytoplasm、nucleus、chloroplasts、endoplasmic reticulum、mitochondria plasma membrane |
| VvANS109 | 28.78% | 55.23% | 15.99% | cytoplasm、nucleus、chloroplasts、mitochondria 、peroxisome |
| VvANS110 | 35.51% | 48.91% | 15.58% | chloroplasts、nucleus、mitochondria、extracellular、vacuole、endoplasmic reticulum |
| VvANS111 | 36.87% | 45.25% | 17.88% | nucleus、cytoplasm、cytoskeleton、plasma membrane、chloroplasts |
| VvANS112 | 42.86% | 40.43% | 16.72% | nucleus、cytoplasm、extracellular |
| VvANS113 | 30.03% | 51.70% | 18.27% | nucleus、chloroplasts、Golgi apparatus plasma membrane |
| VvANS114 | 31.93% | 49.70% | 18.37% | cytoplasm、nucleus、chloroplasts、plasma membrane、Golgi apparatus |
| VvANS115 | 29.23% | 51.37% | 19.40% | cytoplasm、nucleus、extracellular、cytoskeleton |
| VvANS116 | 26.47% | 58.02% | 15.51% | nucleus、chloroplasts、cytoplasm、cytoskeleton |
| VvANS117 | 31.62% | 48.72% | 19.66% | chloroplasts、nucleus、plasma membrane |
| VvANS118 | 30.05% | 50.27% | 19.67% | cytoskeleton 、cytoplasm、extracellular、nucleus |
| VvANS119 | 30.60% | 50.00% | 19.40% | cytoskeleton、extracellular、cytoplasm |
| VvANS120 | 34.08% | 49.50% | 16.42% | cytoplasm、chloroplasts、mitochondria  、plasma membrane、nucleus、peroxisome、cytoskeleton |
| VvANS121 | 25.85% | 53.17% | 20.98% | cytoplasm、chloroplasts、cytoplasm endoplasmic reticulum、nucleus、cytoskeleton |
