# Supplementary material for: Genome-wide identification of grape ANS gene family and expression analysis at different fruit coloration stages
Source: BMC Plant Biol. 2023 Dec 9;23:632. doi: 10.1186/s12870-023-04648-3 (PMC10709965; doi:10.1186/s12870-023-04648-3)
Supplement: Supplementary file 4 — Additional file 4: Supplementary Table S4. qRT-PCR primers for expression on analysis of VvANS gene. [file 12870_2023_4648_MOESM4_ESM.docx]

**Supplementary Table S4.** qRT-PCR primers for expression on analysis of *VvANS* gene

| gene | Forward primer(5’-3’) | Reverse primer (5’-3’) |
| --- | --- | --- |
| *VvANS3* | CAGATACCCTCCTTGTCCCCTACC | AATTCCGATCCACTGGTTGTCACTC |
| *VvANS4* | TGTCCAGGCGTCAGATGTCTCC | ATCGTGTTGGCAGGCTTGGC |
| *VvANS8* | GGTGGGTGAAGAGATGAGCAAAGAG | AAGGGGTTGTTGGAAGCAAGAGC |
| *VvANS9* | GGTGGGTGAAGAGATGAGCAAAGAG | AAGGGGTTGTTGGAAGCAAGAGC |
| *VvANS12* | TGGGGCTTCTTTCAAGTCATCAACC | TCACCTTCCTCTTCTCCTCCAATGG |
| *VvANS13* | GCTCCGTCCGCTTCAATCACTAC | TCCAACCCTCCGACATCATCCTG |
| *VvANS17* | AACTTCATCCGCCTCAATCACTACC | CTTCCAACCCTCCAACATCATCCTG |
| *VvANS20* | AAGAGGAAGGTGAAGAGGGATGAGG | CTTGAGGGTCTGGAGAGGCTGAG |
| *VvANS21* | CGAGCCCAAGAACTAGCCCAAC | CAACATCCTTCACACCATGCACAAC |
| *VvANS22* | TCCCAAGTTCCCAAGCGTCAAATC | AGGATCAGTGGTGAAGGTGTAGGAG |
| *VvANS24* | CTGCTGCGTCCGAAGGCTATG | ACGAGAAAGAGGCAAGGTGTGATG |
| *VvANS25* | CTGCTGCGTCCGAAGGCTATG | ACGAGAAAGAGGCAAGGTGTGATG |
| *VvANS26* | CTGGACTTCACCTCCGTTCATTCTC | GCCAACAAGCTGAGCAGCATTG |
| *VvANS27* | ACTTCTCCTACCCACTACGAACCAG | TCAGACAACACCTCCAGCAACTTG |
| *VvANS28* | CCGTGCTACAGAGGTTGGATTGG | AGCAGCAAACCGATCAGAATGGG |
| *VvANS31* | CTTCAGTCTTCAGCAGCCTCATCAC | GCTCTCCAAATCCAATGTTGTTGCC |
| *VvANS33* | CATCCGTCCAGCCATCCTCAAAG | CATAGTCGCTCACTGTGGTTTCCC |
| *VvANS35* | CAATGATCCAATTCGCCGGAACAAG | CCATCATCTCCTCCAACACGCTAAC |
| *VvANS40* | GAGGCAGAAATCGGATCGCAAAATG | CATCCACGAGTCCCTTTACACCAAG |
| *VvANS43* | AGCATCTTCCACAGAGTCCTAGCC | TCCTTAATGGGTCCGTAGAGTCTCG |
| *VvANS45* | CAAACCAGACCACCTAAGAGCCATG | CAGTGTGCTTGCTAGTGCCCATAG |
| *VvANS46* | AGGTGGCTGAACTGATATGTGATGC | AGGCAACGCAAAGAACCGATGAG |
| *VvANS47* | GCGAGAACTCTCTTTGAGGCTATGG | TGGCTTGCATGGTCACGATTAGG |
| *VvANS50* | ATTGGGCAATGGCTGGGTGTC | TCGTCCGTGCTTCTCTTGGATTTG |
| *VvANS53* | CGACCGAATGAAGGAAGTGAAGGAG | GGGTTTGAGTTGGGAGAGAGTTTGG |
| *VvANS55* | ACCAGAATGGAGTGAAGCACCTTTG | GGGTCGTTCAGAGACAGGCAATATG |
| *VvANS58* | AATACGAACGAAGGATGGCGAGTG | GATGACCCGATGCTCAGGAGAATG |
| *VvANS60* | TTTGAAGCCTGGGTCAGATTCTTGG | TGCCATGAAACAGCCGTACTCTTC |
| *VvANS63* | GCAGGGATGGTGATGGTAGTGAAG | AAAGCAGCCCCATGAGGAAAGC |
| *VvANS64* | GGGTTGATGCCACACACTGACAG | AGTCGCCAATGTTGATGATGAGAGC |
| *VvANS66* | GGAGAGATGGAAGGGTTGGTTGATG | GTGGCGTCGGAATGAGGTGTAAG |
| *VvANS67* | TGATGTTGAAGGGTATGGGCTTTCC | GCTCTGGCAATAGATACGGCTTCC |
| *VvANS71* | CAACCGGCATCACCATCCTTCTC | GCTCACAGGAATCCAAACCCCATC |
| *VvANS73* | AACCAAAGTTCGTCCTCGGTATGTG | GTGAAGCCTCTCCAACTCAGAATCC |
| *VvANS76* | CCATCGAGCATCGTGCGGTTG | GAATGTGGAGTGACAAGGCTAGGC |
| *VvANS77* | TCTTGCCATGACAATCCTTGGGTTC | CCTCATTGCCTGATGCCCTTCTTC |
| *VvANS79* | TGGCAGACAAGAATGGAAGCAGAC | TTGGGAGCTGGTGAAATGATGGC |
| *VvANS82* | TCCTCTCCTCTTCGTCGCTATCTTC | CGCCTGAAAGCAGCCCCATG |
| *VvANS84* | TCCTCTCCTCTTCGTCGCTATCTTC | CGCCTGAAAGCAGCCCCATG |
| *VvANS88* | TGGACTTGCTGTGTGAGAATCTTGG | GGCTTGGGGCAGGGAGGATAG |
| *VvANS90* | TGCGAGTTCAATCACCGTACTTCTG | TCCCGTTATTCCAAGCCTCAATAGC |
| *VvANS91* | CGACAACTTGGGCACAGACTGG | ACTCTGGCTCGGGACAAGGTG |
| *VvANS96* | GTTGCTTCACCATCCTTCTCCAGAG | CCCACATGAACTTGTAGGGCACTG |
| *VvANS100* | TCTTCGGCACCAATCCAACATTCC | GCAGCAGGGTCTCCAGAGAGG |
| *VvANS101* | AGGAACACTGGTGGTGAACATTGG | AACACAACTCGGTGCTCAGATGATC |
| *VvANS108* | GCCTCCTTCTGCCATTAACTACCAC | AAGAGCCTGTCCGCCACTCC |
| *VvANS110* | TACTGACAAATGGGCGGTACGAAAG | GGTGGTCCATGTCCTGTTGCTATG |
| *VvANS113* | TTGGATCAAGCGGCGATATTGGTC | CTCACAAGCCATCTTCCTCACAGC |
| *VvANS119* | GCAGCCGAGAGGGTTGGTTTC | CCTTCCGATACACCGCCTTCTTC |
| *VvANS120* | TATTTCAGGCACCAGCAGCTACTTG | CATACCGCAGGCATATCTGTTGGG |
| *VvGAPDH* | TTCTCGTTGAGGGCTATTCCA | CCACAGACTTCATCGGTGACA |
